# Long-acting methylphenidate formulations in the treatment of attention-deficit/hyperactivity disorder: a systematic review of head-to-head studies

**DOI:** 10.1186/1471-244X-13-237

**Published:** 2013-09-27

**Authors:** David Coghill, Tobias Banaschewski, Alessandro Zuddas, Antonio Pelaz, Antonella Gagliano, Manfred Doepfner

**Affiliations:** 1 Division of Neuroscience, Medical Research Institute, Ninewells Hospital and Medical School, Dundee DD1 9SY, UK; 2 Department of Child and Adolescent Psychiatry, Central Institute of Mental Health, Medical Faculty of Mannheim, University of Heidelberg, Mannheim, Germany; 3 Department of Biomedical Sciences, University of Cagliari, Cagliari, Sardinia, Italy; 4 Department of Child and Adolescent Psychiatry, Hospital Clinico Universitario San Carlos, Madrid, Spain; 5 Department of Pediatric Science, University of Messina, Policlinico Universitario G. Martino, Messina, Italy; 6 Department of Child and Adolescent Psychiatry, University of Cologne, Cologne, Germany

**Keywords:** Methylphenidate, Attention-deficit/hyperactivity disorder, Comparison, Long-acting formulation, Pharmacokinetics, Review

## Abstract

**Background:**

The stimulant methylphenidate (MPH) has been a mainstay of treatment for attention-deficit/hyperactivity disorder (ADHD) for many years. Owing to the short half-life and the issues associated with multiple daily dosing of immediate-release MPH formulations, a new generation of long-acting MPH formulations has emerged. Direct head-to-head studies of these long-acting MPH formulations are important to facilitate an evaluation of their comparative pharmacokinetics and efficacy; however, to date, relatively few head-to-head studies have been performed.

The objective of this systematic review was to compare the evidence available from head-to-head studies of long-acting MPH formulations and provide information that can guide treatment selection.

**Methods:**

A systematic literature search was conducted in MEDLINE and PsycINFO in March 2012 using the MeSH terms: attention deficit disorder with hyperactivity/drug therapy; methylphenidate/therapeutic use and All Fields: Concerta; Ritalin LA; OROS and ADHD; Medikinet; Equasym XL and ADHD; long-acting methylphenidate; Diffucaps and ADHD; SODAS and methylphenidate. No filters were applied and no language, publication date or publication status limitations were imposed. Articles were selected if the title indicated a comparison of two or more long-acting MPH preparations in human subjects of any age; non-systematic review articles and unpublished data were not included.

**Results:**

Of 15,295 references returned in the literature search and screened by title, 34 articles were identified for inclusion: nine articles from pharmacokinetic studies (nine studies); nine articles from laboratory school studies (six studies); two articles from randomized controlled trials (two studies); three articles from switching studies (two studies) and three articles from one observational study.

**Conclusions:**

Emerging head-to-head studies provide important data on the comparative efficacy of the formulations available. At a group level, efficacy across the day generally follows the pharmacokinetic profile of the MPH formulation. No formulation is clearly superior to another; careful consideration of patient needs and subtle differences between formulations is required to optimize treatment. For patients achieving suboptimal symptom control, switching long-acting MPH formulations may be beneficial. When switching formulations, it is usually appropriate to titrate the immediate-release component of the formulation; a limitation of current studies is a focus on total daily dose rather than equivalent immediate-release components. Further studies are necessary to provide guidance in clinical practice, particularly in the treatment of adults and pre-school children and the impact of comorbidities and symptom severity on treatment response.

## Background

Attention-deficit/hyperactivity disorder (ADHD) is the most common neurobehavioural disorder in childhood, affecting approximately 5% of children worldwide and persisting into adulthood in a majority of cases [1,2]. Stimulant medication, including methylphenidate (MPH), is a mainstay of treatment for ADHD in children, adolescents and adults [2,3]. Owing to the short half-life of MPH and the varied issues associated with multiple daily dosing of immediate-release MPH formulations (for example, social stigma, reduced compliance, inconvenience and security issues associated with controlled substances in the school or workplace), a new generation of long-acting MPH formulations has emerged (see Table 1 for a summary of MPH formulations and synonyms) [4].

**Table 1:** Summary of long-acting methylphenidate (MPH) formulations

**MPH formulation**	**Synonyms**	**Product availability**	**Modified-release technology**	**Immediate-release: extended-release ratio (%)**	**Duration of action**^**a**^
Biphentin^®^[10]	MPH ER	Canada	Multilayer-release (MLR) bead formulation	40:60	Not stated: biphasic delivery profile
Concerta^®^[12]	Concerta extended release; Concerta^®^ LP; methylphenidate hydrochloride; OROS MPH	Africa^b^, Asia^c^, Australia, Europe^d^, New Zealand, North America^e^, South America^f^	OROS^®^ (Osmotic Release Oral System)	22:78	12 hours
Daytrana^®^[13]	MPH transdermal system; MethyPatch; MTS	USA	Transdermal patch	Continuous delivery	9 hours
Equasym XL^®^[11]	Equasym Depot^®^; Equasym Retard^®^, Equasym XR^®^, Quasym LP^®^; Metadate CD^®^; Metadate ER^®^	Europe^g^, South Korea, USA	Diffucaps^®^	30:70	8 hours
Focalin XR^®^[7]	d-MPH-ER	Switzerland, USA^h^	SODAS^®^ (Spheroidal Oral Drug Absorption System)	50:50	Not stated: two distinct peaks approximately 4 hours apart
Medikinet^®^ retard [9]	Medikinet^®^; Medikinet^®^ CR; Medikinet^®^ EM; Medikinet^®^ MR; Medikinet^®^ XL	Europe^i^, Israel, Korea, South America^j^	Modified-release capsules	50:50	8 hours
Ritalin LA^®^[6]	Ritalin LP^®^; Miacalcic	France, Chile, USA^h^	SODAS^®^ (Spheroidal Oral Drug Absorption System)	50:50	Not stated: two distinct peaks approximately 4 hours apart
Ritalin SR^®^[5]	n/a	Canada, USA^h^	Wax matrix	Continuous release	8 hours

^a^Duration of action as stated in the prescribing information.

^b^Botswana, Brazil, Egypt, Namibia, Nicaragua, South Africa.

^c^Bahrain, China, Hong Kong, Indonesia, Israel, Japan, Jordan, Republic of Korea, Kuwait, Lebanon, Malaysia, Oman, Philippines, Qatar, Saudi Arabia, Singapore, Taiwan Province of China, United Arab Emirates, Yemen.

^d^Austria, Belgium, Croatia, Czech Republic, Denmark, Estonia, Finland, France, Germany, Greece, Iceland, Ireland, Latvia, Lichtenstein, Lithuania, Luxembourg, Malta, Netherlands, Norway, Poland, Portugal, Romania, Slovakia, Slovenia, Spain, Sweden, Switzerland, UK.

^e^Bahamas, Barbados, Canada, Cayman Islands, Dominican Republic, El Salvador, Guatemala, Honduras, Jamaica, Mexico, Trinidad and Tobago, USA.

^f^Argentina, Aruba, Bolivia, Chile, Columbia, Costa Rica, Ecuador, Panama, Paraguay, Peru, Uruguay, Venezuela.

^g^Denmark, Finland, France, Germany, Ireland, the Netherlands, Norway, Sweden, Switzerland, UK.

^h^Information obtained from Thomson Cortellis™.

^i^Austria, Belgium, Cyprus, Denmark, Estonia, Finland, France, Germany, Ireland, Italy, Latvia, Lithuania, Luxembourg, the Netherlands, Norway, Poland, Portugal, Romania, Slovakia, Slovenia, Spain, Sweden, Switzerland, UK.

^j^Argentina, El Salvador, Guatemala, Honduras.

Most of these newer long-acting MPH formulations differ from the first-generation, wax-matrix, continuous-release preparation (Ritalin SR^®^; Novartis Pharmaceutical Corporation, East Hanover, New Jersey, USA [5]) by including an immediate-release component that ensures a rapid onset of action as well as an extended-release component that continues to act throughout the course of the day. This allows for rapid onset of action with once-daily dosing while avoiding the need to take a second dose of medication during the school or work day. The various MPH formulations use different technologies that aim to provide symptom control for at least 8 hours and also incorporate differing proportions of immediate- and extended-release MPH. As a consequence, the immediate-release MPH bolus of the new formulations ranges from 22 to 50% of the total MPH dose. Ritalin LA^®^ and Focalin XR^®^ (both Novartis Pharmaceutical Corporation, East Hanover, New Jersey, USA) use Spheroidal Oral Drug Absorption System (SODAS^®^) technology to provide 50% of the MPH dose immediately and 50% as extended release [6,7]. Like most MPH formulations, Ritalin LA^®^ contains racemic MPH comprising both the *d-*MPH and the *l-*MPH isomers; however, as when taken orally the *l*-isomer is metabolized rapidly via first pass through the hepatic circulation it is considered that the *d-*isomer is likely to be the main pharmacological contributor to efficacy in the treatment of ADHD [8]. Focalin XR^®^ contains only the *d-*MPH isomer and can, therefore, achieve similar efficacy at a lower total daily dose than the racemic MPH formulations [8]. Medikinet^®^ retard (MEDICE Pharma GmbH and co. KG, Iserlohn, Germany) also provides 50% of the racemic MPH dose immediately, using a 50:50 mixture of immediate release and enteric-coated beads to delay MPH delivery [9]. Biphentin^®^ (Purdue Pharma, Pickering, Ontario, Canada) uses a multilayer-release bead formulation to provide a rapid initial release of 40% of the total racemic MPH dose followed by delivery of the remaining MPH from a controlled-release core [10]. Equasym XL^®^ (Metadate CD^®^; Shire Pharmaceuticals Ireland Ltd, Dublin, Ireland) employs the Diffucaps^®^ bead delivery technology to deliver 30% of the racemic MPH immediately and 70% from extended-release beads, while Concerta^®^ (Janssen-Cilag Ltd, High Wycombe, UK) uses the osmotic controlled-release delivery system (OROS^®^) to release 22% of racemic MPH immediately followed by gradual delivery of the remaining MPH throughout the day [11,12]. Continuous delivery of racemic MPH is provided by Daytrana^®^ (Noven Pharmaceuticals Inc., Miami, Florida, USA) via the MPH transdermal system, a diffusion-based patch applied to the skin [13].

The differing time–action profiles provided by these long-acting MPH formulations may allow clinicians to target specific periods of the day that are particularly relevant for a patient, facilitating individualization of ADHD treatment.

Response to MPH in the treatment of ADHD varies between patients. While MPH is effective in the majority of children in the short term, there is significant variation in individual response to treatment, with a minority not achieving adequate symptom control and others unable to tolerate MPH due to adverse effects [14-16]. Optimization of dose and treatment regimen is needed, therefore, and continued monitoring of response throughout the treatment period is required [16]. Given the range of MPH formulations available and the individualization of therapy that is required to ensure optimal treatment, direct head-to-head studies of long-acting MPH formulations can provide important information about the comparative pharmacokinetics (PK), pharmacodynamics and efficacy of the different formulations. While a number of studies compare long- and short-acting MPH formulations, for example Medikinet^®^ retard versus twice-daily immediate-release MPH [17], few direct head-to-head studies of two or more long-acting MPH formulations have been performed to date.

The objective of this review was to bring together the evidence available from head-to-head studies of long-acting MPH formulations and provide evidence-based clinical guidance on treatment selection.

## Methods

A literature search was conducted using the MEDLINE (1950–Present) and PsycINFO (1806–Present) databases to identify head-to-head studies of long-acting MPH formulations. The final search was conducted on 21 March 2012 and followed the PRISMA (Preferred Reporting Items for Systematic Reviews and Meta-Analyses) guidelines for methodology. The following search terms were developed, refined and tested for relevance by cross-checking results against a list of known relevant articles: MeSH terms: attention deficit disorder with hyperactivity/drug therapy; methylphenidate/therapeutic use; All Fields: Concerta; Ritalin LA; OROS and ADHD; Medikinet; Equasym XL and ADHD; long-acting methylphenidate; Diffucaps and ADHD; SODAS and methylphenidate. In addition, searches for Biphentin and ADHD, methylphenidate transdermal system and ADHD, and Metadate CD and ADHD were performed following the final search to corroborate findings (no additional studies were identified that met the search criteria). No filters were used during the search as key references were missed during the development and testing of the search strategy when filters were applied. No language, publication date or publication status limitations were imposed. All search results were combined into a single master database and duplicates removed. References within the master database were then screened by title for the following keywords to identify articles of possible relevance: methylphenidate; Concerta; Ritalin LA; OROS; Medikinet; Equasym XL; Diffucaps; SODAS; Spheroidal Oral Drug Absorption System; extended-release; extended release; long-acting; long acting; modified-release; modified release; single-dose; single dose; once-daily; once daily; methylphenidate transdermal system; MTS; osmotic-release; osmotic release; laboratory; meta-analysis; once-a-day; once a day. Articles were selected if the title indicated a comparison of two or more long-acting methylphenidate preparations in humans; articles with ambiguous titles were selected for further screening by abstract and/or full text. Studies involving subjects of any age with ADHD of any subtype receiving long-acting MPH were considered. Study diagnostic criteria and inclusion criteria were not assessed during the screening process. Both switching studies and observational studies were included; however, non-systematic review articles and non-peer-reviewed data were not included in this review. Articles selected by title screening were then assessed independently for eligibility by a second person; potentially eligible articles were then screened by abstract and disagreement regarding eligibility was resolved through discussion. If the suitability of an article was unclear, the full-text article was assessed. Reference lists of relevant systematic reviews and meta-analyses were cross-referenced against identified articles. Data extraction, performed independently by two people, included article category, drug and dosing, age group, sample size, diagnosis, inclusion/exclusion criteria, comorbid conditions, outcome measures, main findings and conclusions. Possible sources of bias were identified as multiple reports from single studies and variability in outcome measures and parameters among studies. Meta-analysis was not feasible owing to the heterogeneity in outcomes reported across all study types included in the review; however, a systematic approach was applied to identify comparisons of interest and synthesize the findings of multiple studies.

## Results and discussion

**Figure 1:**
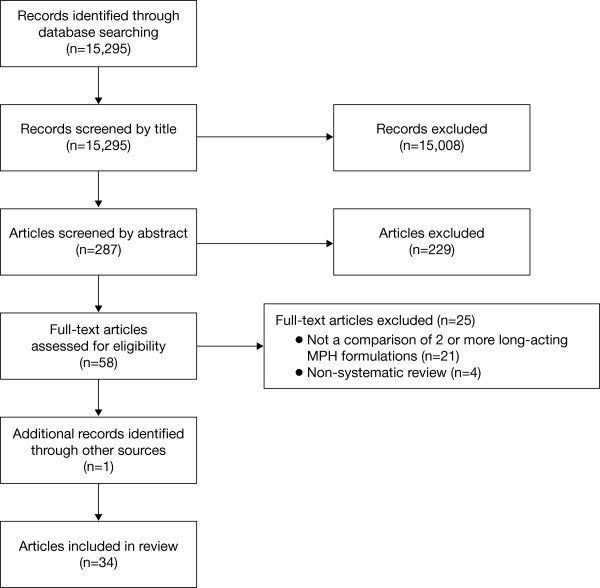
Flow diagram of screened and included articles.

The master database included 15,295 references, which were screened by title for relevant articles (Figure 1). Of these, 287 articles were screened by abstract and 33 articles were identified for inclusion in the review. One additional article, which was not in the master database (Silva et al., 2008 [18]), was identified from the reference list of a systematic review (Brams et al., 2010 [19]). Publications included in the review are summarized in Table 2 and comprised nine articles from PK studies (nine studies); nine articles from laboratory school studies (six studies); two articles from randomized controlled trials (RCTs; two studies); three articles from switching studies (two studies) and three articles from one observational study. As the studies were diverse and the reported outcomes were heterogeneous, consistent comparisons of interest could not be made across all study types. For PK articles (n = 9), comparisons of interest were bioequivalence of long-acting MPH formulations to Concerta^®^, overall MPH exposure, time to peak plasma MPH concentration, plasma MPH concentrations across the day, and the effect of long-acting MPH formulations on dopamine transport occupancy. Comparisons of interest for laboratory school study articles (n = 9) were efficacy of long-acting MPH formulations across the day, and the effect of symptom severity and gender on MPH response. For RCTs, switching and observational study articles, similarities and differences between outcomes relating to different long-acting MPH formulations are reported, as available. An overview of the main conclusions from eight systematic reviews and meta-analyses addressing long-acting ADHD medications, including MPH, are also presented. Effect sizes were reported in only five articles from two studies (excluding meta-analyses); therefore, effect sizes are presented in the text where appropriate but are not included in Table 2.

**Table 2:** Summary of articles identified in the literature search that compare ≥2 long-acting MPH formulations

**Author, year**	**Long-acting MPH formulation and dosing**^**a**^	**Sample size Age range**	**Diagnosis**	**Comorbid conditions**	**Outcome measures**
**Pharmacokinetic studies**					
Gonzalez et al., 2002 [20]	Concerta^®^ (18, 36, 54 mg)	n = 36	Not applicable	Not stated	PK parameters for 24 hours post-dose, including bioequivalence
	Equasym XL^®^ (20, 40, 60 mg)	21–40 years			
Haessler et al., 2008 [26]	Ritalin LA^®^ (40 mg)	n = 28	Not applicable	Not stated	PK parameters for 24 hours post-dose, including bioequivalence
	Medikinet^®^ retard (40 mg)	18–30 years			
Markowitz et al., 2003 [22]	Concerta^®^ (18 mg)	n = 20	Not applicable	Not stated	PK parameters for 24 hours post-dose, including bioequivalence
	Ritalin LA^®^ (20 mg)	21–34 years			
Modi et al., 2000 [23]	Concerta^®^ (18 mg)	n = 36	Not applicable	Not stated	PK parameters for 30 hours post-dose
	Ritalin SR^®^ (20 mg)	18–41 years			
Pierce et al., 2010 [24]	Concerta^®^ (18, 27, 36, 54 mg)	n = 71	ADHD according to DSM-IV-TR criteria	Possibly ODD	PK properties of *d*,*l*-MPH after single, multiple, fixed and escalating doses of Concerta^®^ and Daytrana^®^
	Daytrana^®^ (10, 15, 20, 30 mg/9 hours)	6–17 years			
Reiz et al., 2008 [25]	Concerta^®^ (18 mg)	n = 24	Not applicable	Not stated	PK parameters for 24 hours post-dose, including bioequivalence
	Biphentin^®^ (20 mg)	19–25 years			
Schutz et al., 2009 [28]	Equasym XL^®^ (20 mg)	n = 14	Not applicable	Not stated	PK parameters for 24 hours post-dose, including bioequivalence
	Medikinet^®^ retard (20 mg)	22–43 years			
Spencer et al., 2010 [21]	Concerta^®^ (36 mg)	n = 21	Not applicable	Not stated	PET imaging
	Equasym XL^®^ (40 mg)	18–55 years			
Tuerck et al., 2007 [27]	Focalin XR^®^ (20 mg)	n = 25	Not applicable	Not stated	PK parameters for 24 hours post-dose, including bioequivalence
	Ritalin LA^®^ (40 mg)	19–45 years			
**Laboratory school studies**					
Lopez et al., 2003 [31]	Concerta^®^ (18, 36 mg)	n = 36	ADHD according to C-DISC criteria	Not stated	SKAMP-Attention; SKAMP-Deportment; SKAMP-Combined; Math-Attempted; Math-Correct
	Ritalin LA^®^ (20 mg)	6–12 years			
Muniz et al., 2008 [33]	Concerta^®^ (36, 54 mg)	n = 84	Combined-type ADHD (89%); Inattentive-type ADHD (11%) according to DSM-IV criteria, established by C-DISC	Not stated	SKAMP-Combined; SKAMP-Attention; SKAMP-Deportment; Math-Attempted; Math-Correct; CPRS
	Focalin XR^®^ (20, 30 mg)	6–12 years			
Schulz et al., 2010 [38]	Ritalin LA^®^ (20 mg)	n = 147	Combined-type ADHD (55%); Inattentive-type ADHD (37%); Hyperactive/impulsive-type ADHD according to DSM-IV criteria, confirmed by K-SADS	Disturbance in social behaviour (n = 4), initial insomnia (n = 2), ODD (n = 2), dysphemia (n = 1), encopresis (n = 1)	SKAMP-Combined; Math-Attempted; Math-Correct; NCBRF-TIQ
	Medikinet^®^ retard (20 mg)	6–14 years			
Silva et al., 2005 [32]	Concerta^®^ (18 mg)	n = 54	Combined inattentive/hyperactive-type ADHD (70%); Inattentive-type ADHD (28%); Hyperactive/impulsive-type ADHD (2%) according to DSM-IV criteria	Not stated	SKAMP-Attempted; SKAMP-Deportment; SKAMP-Combined; Math-Attempted; Math-Correct
	Ritalin LA^®^ (20 mg)	6–12 years			
Silva et al., 2008 [18]	Concerta^®^ (36, 54 mg) Focalin XR^®^ (20, 30 mg)	n = 82 6–12 years	Combined-type ADHD (94%); Inattentive-type (6%) according to DSM-IV criteria	Not stated	SKAMP-Attention; SKAMP-Deportment; SKAMP-Combined; Math-Attempted; Math-Correct; CPRS
	
Sonuga-Barke et al., 2004 [34]	Concerta^®^ (18, 36, 54 mg) Equasym XL^®^ (20, 40, 60 mg)	n = 184 6–12 years	Combined-type (82%); Inattentive-type (13%); Hyperactive/impulsive-type (5%) according to DSM-IV criteria and confirmed by DISC	Comorbid condition (25%), including anxiety and ODD	Placebo-adjusted SKAMP-Combined
Post-hoc study of COMACS [37]					
Sonuga-Barke et al., 2007 [35]	Concerta^®^ (18, 36, 54 mg) Equasym XL^®^ (20, 40, 60 mg)	n = 184 6–12 years	Females: Combined-type (77%); Inattentive-type (15%); Hyperactive/impulsive-type (8%). Males: Combined-type (84%); Inattentive-type (12.4%); Hyperactive/impulsive-type (4%)	Comorbid condition (25%), including anxiety and ODD	SKAMP-Combined; PERMP
Post-hoc study of COMACS [37]					
Sonuga-Barke et al., 2008 [36]	Concerta^®^ (18, 36, 54 mg)	n = 184	Combined-type (82%); Inattentive-type (13%); Hyperactive/impulsive-type (5%) according to DSM-IV criteria, confirmed by DISC	Comorbid condition (25%), including anxiety and ODD	GMM analysis
	Equasym XL^®^ (20, 40, 60 mg)	6–12 years			
Post-hoc study of COMACS [37]					
Swanson et al., 2004 [37]	Concerta^®^ (18, 36, 54 mg)	n = 184	Combined-type (82%); Inattentive-type (13%); Hyperactive/impulsive-type (5%) according to DSM-IV criteria, confirmed by DISC	Comorbid condition (25%), including anxiety and ODD	SKAMP-Attention; SKAMP-Deportment; PERMP
	Equasym XL^®^ (20, 40, 60 mg)	6–12 years			
COMACS study					
**Randomized controlled trials**					
Doepfner et al., 2011 [42]	Concerta^®^ (18, 36 mg)	n = 113	Combined-type ADHD according to DSM-IV, confirmed by interview (DCL-ADHD)	ODD or conduct disorder (36%)	SKAMP-D; DAYAS; FBB-ADHD
	Medikinet^®^ retard (10, 20, 30 mg)	6–16 years			
Findling et al., 2008 [43]	Concerta^®^ (18, 27, 36, 54 mg)	n = 282	Combined-type ADHD (71–86%); Inattentive-type ADHD (11–26%); Hyperactive/impulsive-type ADHD (1–2%) according to DSM-IV-TR criteria	Possibly ODD	ADHD-RS-IV mean total score; CTRS-R; CPRS-R; CGI–S; CGI–I; PGA; MPH plasma concentrations at 7.5, 9 and 10 hours post-dose
	Daytrana^®^ (10, 15, 20, 30 mg)	6–12 years			
**Switching studies**					
Arnold et al., 2010 [46]	Concerta^®^ (18, 27, 36, 45, 54 mg)	n = 171	Combined-type ADHD (77%); Inattentive-type ADHD (21%); Hyperactive/impulsive -type ADHD (2%) according to DSM-IV-TR criteria	Possibly ODD	ADHD-RS-IV mean total scores; CGI–I; PGA, CPRS-R; CGI–S
	Ritalin LA^®^ (10, 20, 30, 40, 50 mg)	6–12 years			
	Equasym XL^®^ (10, 15, 20, 30, 40, 50 mg)				
	Daytrana^®^ (10, 15, 20, 30 mg)				
Bukstein et al., 2009 [47]	Concerta^®^ (18, 27, 36, 45, 54 mg)	n = 171	See Arnold et al., 2010 [46]	Possibly ODD	AIM-C; MSS
	Ritalin LA^®^ (10, 20, 30, 40, 50 mg)	6–12 years			
	Equasym XL^®^ (10, 15, 20, 30, 40, 50 mg)				
	Daytrana^®^ (10, 15, 20, 30 mg)				
Dirksen et al., 2002 [48]	Equasym XL^®^ (20, 40, 60 mg)	n = 308	ADHD according to DSM-IV criteria (diagnostic code 314.01)	Not stated	CGI–I; CGI–S; CGI-Efficacy Index
	Concerta^®^ (18, 36, 54, 72 mg)	6–17 years			
	Other long-acting MPH (excluding Concerta^®^; dose not stated)				
**Observational studies**					
Doepfner et al., 2011 [49]	Equasym XL^®^ (10–120 mg)	n = 822	Disturbance of activity/attention (F90.0; 55%); hyperkinetic conduct disorder (F90.1; 36%); other hyperkinetic disorders (F90.8; 8%) according to ICD-10 criteria	Not stated	CGI–S; CGI–I; FBB-ADHD; DAYAS; SDQ-P
	Other long-acting MPH (most commonly Medikinet^®^ retard; approximately 0.85 mg/kg/day)	6–17 years			
OBSEER study					
Doepfner et al., 2011 [50]	Equasym XL^®^ (10–120 mg)	n = 782	For total study sample (n = 822) see Doepfner et al., 2011 [49]	Not stated	FBB-ADHD; CGI–S; DAYAS; KINDL
	Other long-acting MPH (mean [SD] 29.2 [11.28] mg)	6–17 years			
Post-hoc study of OBSEER [49]					
Rothenberger et al., 2011 [51]	See Doepfner et al., 2011 [49]	n = 822 6–17 years	See Doepfner et al., 2011 [49]	Not stated	KINDL; SAMS
Post-hoc study of OBSEER [49]					
**Meta-analyses**					
Faraone et al., 2006 [55]	Equasym XL^®^; Ritalin LA^®^;	n = 29 articles			Effect size expressed as SMD
	Concerta^®^; Daytrana^®^	Children and adolescents			
Faraone and Buitelaar, 2010 [56]	Equasym XL^®^; Ritalin LA^®^;	n = 23 articles			Effect size expressed as SMD
	Concerta^®^; Daytrana^®^	Children and adolescents			
Faraone and Glatt, 2010 [57]	Concerta^®^;	n = 18 articles			Effect size expressed as SMD
	Focalin XR^®^	Adults			
Peterson et al., 2008 [58]	Concerta^®^;	n = 22 articles			Ratio of relative risks
	Focalin XR^®^	Adults			
**Systematic reviews**					
Banaschewski et al., 2006 [52]	Concerta^®^; Ritalin LA^®^; Equasym XL^®^; Medikinet^®^ retard	Not stated			Effect size expressed as SMD
Brams et al., 2008 [53]	Concerta^®^; Daytrana^®^; Focalin XR^®^; Equasym XL^®^; Ritalin LA^®^	n = 18 articles			SKAMP, CADS-T, IOWA Conners’ Rating Scale, ADHD-RS-IV, PERMP, CGIS-T
		Children and adolescents			
Brams et al., 2010 [19]	Concerta^®^; Focalin XR^®^; Equasym XL^®^; Ritalin LA^®^	n = 15 articles			PERMP
		Children, adolescents and adults			
Swanson et al., 2002 [54]	Concerta^®^; Equasym XL^®^; Ritalin LA^®^	Not stated			SKAMP, 10-Minute Math Test, PK measures, effect size

**ADHD**, attention deficit hyperactivity disorder; **ADHD-RS-IV**, ADHD Rating Scale-version IV; **AIM-C**, ADHD Impact Module-Children; **C-DISC**, Diagnostic Interview Schedule for Children 1997; **CADS-T**, Conners’ ADHD/DSM-IV Scale for teachers; **CGI–I**, Clinical Global Impressions–Improvement; **CGI–S**, Clinical Global Impressions–Severity of Illness; **CGIS-T**, Conners’ Global Index Scale for teachers; **CPRS**, Conners’ Parent Rating Scale; **CTRS-R**, Conner’s Teacher Rating Scale-Revised; **DAYAS**, Day Profile of ADHD Symptoms; **DCL**, Diagnostic Checklist for ADHD; **DSM-IV**, Diagnostic and Statistical Manual of Mental Disorders-Fourth Edition; **DSM-IV-TR**, Diagnostic and Statistical Manual of Mental Disorders-Fourth Edition, Text Revision; **FBB-ADHD**, Fremdbeurteilungsbogen für Aufmerksamkeitsdefizit–Hyperaktivitätsstörung (German symptom checklist for attention deficit hyperactivity disorder); **GMM**, Growth Mixture Modelling; **ICD-10**, International Classification of Diseases, version 10; **KINDL**, Kinder Lebensqualitatsfragebogen; **IOWA**, Inattention/Overactivity With Aggression; **K-SADS**, Kiddie Schedule for Affective Disorders and Schizophrenia; **MPH**, methylphenidate; **MSS**, Medication Satisfaction Survey; **NCBRF-TIQ**, Nisonger Child Behaviour Rating Form; **ODD**, oppositional defiant disorder; **PERMP**, permanent product measure of performance; **PET**, Positron Emission Tomography; **PGA**, Parent Global Assessment; **PK,** pharmacokinetic; **SAMS**; Satisfaction with Medication Scale; **SD**, Standard deviation; **SDQ-P**, Strengths and Difficulties Questionnaire for Parents; **SKAMP**, Swanson, Kotkin, Agler, M-Flynn and Pelham Rating Scale; **SKAMP-D**, German version of Swanson, Kotkin, Agler, M-Flynn and Pelham Rating Scale; **SMD**, Standard Mean Difference.

^a^Long-acting stimulants other than methylphenidate have not been included.

### PK studies

In total, nine studies investigated the PK properties of long-acting MPH formulations in head-to-head comparisons. Six studies used Concerta^®^ as a comparator; of these, two studies compared Concerta^®^ with Equasym XL^®^[20,21], while four individual studies compared Concerta^®^ with Ritalin LA^®^[22], Ritalin SR^®^[23], Daytrana^®^[24] and Biphentin^®^[25]. Therefore, this provides head-to-head comparisons of Concerta^®^ with five long-acting MPH formulations. Additional comparisons of long-acting MPH formulations were Ritalin LA^®^ versus Medikinet^®^ retard [26], Ritalin LA^®^ versus Focalin XR^®^[27], and Equasym XL^®^ versus Medikinet^®^ retard [28]. All, apart from one of these studies, were performed in adults. The Concerta^®^ versus Daytrana^®^ study was performed in children and adolescents [24].

#### Head-to-head PK studies of Concerta***^®^*** versus Equasym XL***^®^***, Ritalin LA***^®^***, Ritalin SR***^®^***, Daytrana***^®^*** and Biphentin***^®^***

##### Bioequivalence to Concerta*^®^* and overall MPH exposure

Three studies assessed the bioequivalence of long-acting MPH formulations with Concerta^®^[20,22,25]. In each, bioequivalence was considered present if the 90% confidence interval (CI) ratio of the two MPH formulations under comparison was within 80–125%. Concerta^®^ was not bioequivalent to comparable daily doses of Equasym XL^®^ (maximum concentration [C_max_]_-1_; area under the curve [AUC]_0–4_; AUC_0–6_) or Ritalin LA^®^ (AUC_0–∞_) [20,22]. While Concerta^®^ and Biphentin^®^ were bioequivalent according to AUC_0–t_ (90% CI 105.62–116.41) and AUC_0–∞_ (90% CI 106.25–116.33), they were, however, not bioequivalent according to C_max_ (90% CI 113.85–130.39) [25]. Bioequivalence between Concerta^®^ and Ritalin SR^®^ or Daytrana^®^ was not evaluated [23,24].

While Concerta^®^ (18 mg) had similar overall (24 hour) MPH exposure (AUC) to Equasym XL^®^ (20 mg) and Ritalin LA^®^ (20 mg) [20,22], exposure to MPH over 24 hours was significantly higher for Biphentin^®^ (20 mg) compared with Concerta^®^ (18 mg, p = 0.002) [25]. This was due to a concentration–time profile for Biphentin^®^ that resulted in the delivery of a significantly greater proportion of MPH compared with Concerta^®^ in the first 4 hours post-dose followed by comparable levels of MPH later in the day [25]. Systemic exposure to *d*-MPH during treatment with Daytrana^®^ was higher in children than adolescents [24]. Systemic exposure to *d*-MPH from a single dose of Daytrana^®^ (10 mg/9 hours) was similar to that from a single dose of Concerta^®^ (18 mg) in children, but only 60–80% of that of Concerta^®^ (18 mg) in adolescents [24]. This difference in systemic exposure was attributed to the lower body weight in children compared with adolescents [24]. After multiple escalating doses of Daytrana^®^ in children (final dose of 30 mg/9 hours), systemic exposure to *d*-MPH was 1.4-fold to 1.6-fold higher compared with multiple escalating doses of Concerta^®^ (final dose: 54 mg) [24]. The investigators concluded that higher accumulation of *d*-MPH with Daytrana^®^ compared with Concerta^®^ was a result of continued long-term administration rather than frequency of dosing or changes in clearance, and also that changes in skin permeability resulting from application-site erythema may be a cause of increased absorption of MPH from Daytrana^®^ during multiple dosing [24]. In contrast with the findings in children, systemic exposure to *d*-MPH was similar in adolescents for both Daytrana^®^ and Concerta^®^[24].

##### Time to peak plasma MPH concentration (T_max_)

In head-to-head studies of Concerta^®^ with five long-acting MPH formulations, Concerta^®^ generally reached peak plasma MPH concentration later (5–8 hours post-dose) than the other long-acting MPH formulations investigated (Ritalin LA^®^, Biphentin^®^, Ritalin SR^®^ and Equasym XL^®^; 4–6 hours post-dose) with the exception of Daytrana^®^ which reached peak levels 10 hours post-dose (Table 3) [20,22-25]. T_max_ was not provided by Gonzalez et al. for Equasym XL^®^ versus Concerta^®^ and therefore could not be included in Table 3; however, both MPH formulations displayed biphasic characteristics, providing a sharp initial increase in MPH plasma concentration at approximately 1 hour post-dose and a second peak 6 hours post-dose (Equasym XL^®^) and 6–8 hours post-dose (Concerta^®^) [20].

**Table 3:** Pharmacokinetic parameters across the day for head-to-head pharmacokinetic studies (presented for single-dose comparison only)

	**C**_**max**_**, ng/mL**			**T**_**max**_**, hours**			**AUC, ng • h/mL**		
	**C**_**max0–4**_	**C**_**max4–10**_	**C**_**max**_	**T**_**max0–4**_	**T**_**max4–10**_	**T**_**max**_	**AUC**_**0–4**_	**AUC**_**4–10**_	**AUC**_**0–∞**_
**Gonzalez et al., 2002**[20]**(fasted state)**									
Concerta^®^	–	–	–	–	–	–	6.28	–	36.43
(18 mg)							(2.65)		(13.50)
Equasym XL^®^	–	–	–	–	–	–	10.01	–	39.74
(20 mg)							(3.06)		(11.75)
Concerta^®^	–	–	–	–	–	–	11.88	–	94.05
(36 mg)							(5.86)		(44.51)
Equasym XL^®^	–	–	–	–	–	–	20.36	–	98.49
(2 × 20 mg)							(8.74)		(52.05)
Concerta^®^	–	–	–	–	–	–	18.81	–	143.38
(54 mg)							(7.18)		(64.83)
Equasym XL^®^	–	–	–	–	–	–	32.01	–	145.34
(3 × 20 mg)							(13.09)		(65.21)
**Haessler et al., 2008**[26]**(fed state)**									
Ritalin LA^®^	11.8	12.8	13.3	–	–	–	33.5	57.8	126.8
(40 mg)	(3.95)	(4.13)	(4.04)				(11.07)	(16.91)	(34.5)
Medikinet^®^ retard	16.5	18.3	19.6	–	–	–	42.4	68.0	141.5
(40 mg)	(6.69)	(5.66)	(5.95)				(16.54)	(19.47)	(38.3)
**Haessler et al., 2008**[26]**(fasted state)**									
Ritalin LA^®^	10.0	14.5	14.5	–	–	–	27.6	57.1	114.1
(40 mg)	(3.51)	(3.02)	(3.02)				(8.89)	(14.36)	(30.8)
Medikinet^®^ retard	16.5	14.7	17.1	–	–	–	44.9	49.3	115.7
(40 mg)	(4.62)	(4.45)	(4.88)				(14.77)	(18.47)	(37.2)
**Markowitz et al., 2003**[22]**(fasted state)**									
Concerta^®^	3.4	–	5.9	3.3	–	6.0	9.3	–	66.9
(18 mg)	(44)^a^		(37)^a^	(36)^a^		(28)^a^	(51)^a^		(49)^a^
Ritalin LA^®^	7.0	–	9.9	2.1	–	5.5	18.5	–	78.7
(20 mg)	(47)^a^		(41)^a^	(48)^a^		(15)^a^	(44)^a^		(54)^a^
**Modi et al., 2000**[23]**(fasted state)**									
Concerta^®^	–	–	3.75	–	–	6.7	–	–	42.0
(18 mg)			(1.0)			(1.8)			(14)
Ritalin SR^®^	–	–	4.84	–	–	3.7	–	–	46.7
(20 mg)			(1.6)			(1.6)			(16)
**Pierce et al., 2010**[24]**(fasted state; age 6–12 years)**									
Concerta^®^	–	–	7.80	–	–	6.02	–	–	94.2
(18 mg)			(3.35)			(4.0–10.0)^b^			(43.8)
Daytrana^®^	–	–	9.30	–	–	10.0	–	–	99.2
(10 mg/9 hours)			(3.60)			(8.0–12.0)^b^			(42.9)
**Pierce et al., 2010**[24]**(fasted state; age 13–17 years)**									
Concerta^®^	–	–	4.95	–	–	8.0	–	–	60.1
(18 mg)			(1.42)			(1.0–10.0)^b^			(16.3)
Daytrana^®^	–	–	4.15	–	–	10.0	–	–	48.7
(10 mg/9 hours)			(2.59)			(6.0–12.0)^b^			(21.9)
**Reiz et al., 2008**[25]**(fed state)**									
Concerta^®^	3.33	4.09	4.13	2.51	5.86	4.96	8.77	26.29	48.21
(18 mg)	(1.03)	(1.01)^c^	(1.01)	(1.15)	(1.61)^c^	(2.56)	(3.37)	(5.57)^c^	(9.79)
Biphentin^®^	4.8	4.73	5.07	2.26	6.02	3.71	12.35	28.57	54.01
(20 mg)	(1.39)	(1.09)^c^	(1.32)	(0.64)	(1.26)^c^	(2.03)	(3.78)	(6.68)^c^	(13.11)
**Schutz et al., 2009**[28]**(fed state)**									
Equasym XL^®^	3.82	–	4.05	3.24	–	3.99	9.66	–	37.35
(20 mg)	(0.96)		(0.96)	(1.13)		(1.88)	(3.24)		(10.92)
Medikinet^®^ retard	4.83	–	5.26	2.82	–	4.06	11.72	–	39.90
(20 mg)	(1.87)		(2.11)	(1.00)		(1.65)	(4.64)		(13.77)
**Tuerck et al., 2007**[27]**(fasted state)**									
Focalin XR^®^	13.7	14.9	15.5	1.5	6.5	5.8	36.3	59.1	119
(20 mg)	(4.6)	(4.0)	(4.3)	(1.0–2.0)^b^	(4.5–7.0)^b^	(1.0–7.0)^b^	(10.6)	(16.0)	(40.7)
Ritalin LA^®^	13.2	16.3	16.4	2.0	6.5	6.5	35.0	60.9	122
(40 mg)	(3.0)	(4.5)	(4.4)	(1.5–4.5)^b^	(4.0–8.0)^b^	(2.0–7.0)^b^	(8.7)	(15.0)	(36.3)

Data are shown as mean (standard deviation) unless stated otherwise.

**AUC**, area under the curve; **C**_**max**_, maximum plasma methylphenidate concentration; **T**_**max**_, time to maximum plasma methylphenidate concentration.

^a^Percentage coefficient of variation; ^b^median (range); ^c^4–12 hours post-dose.

N.B. Spencer et al., 2010 [21] did not present the values listed; therefore this reference was not included in the table.

##### Plasma MPH concentrations across the day

Morning: In the first 4 hours post-dose, Equasym XL^®^ (20, 40, 60 mg), Ritalin LA^®^ (20 mg), Ritalin SR^®^ (20 mg) and Biphentin^®^ (20 mg) reached higher plasma MPH concentrations (AUC_0–4_ and C_max0–4_) than comparable daily doses of Concerta^®^ (Table 3) [20,23-25]. Data for AUC_0–4_ and C_max0–4_ for Concerta^®^ versus Daytrana^®^ were not presented by Pierce and colleagues; however, a delay of approximately 2 hours in the absorption of *d*-MPH in children and adolescents following a single dose of Daytrana^®^ (10 mg/9 hours) was reported, which was not apparent in those receiving Concerta^®^, or following multiple fixed or escalating doses of Daytrana^®^[24].

Afternoon and evening: While Equasym XL^®^ (20, 40, 60 mg) produced greater MPH concentrations compared with the nearest daily dose of Concerta^®^ (18, 36, 54 mg) up to 6 hours post-dose, this reversed later in the day, with Concerta^®^ sustaining greater plasma MPH concentrations than Equasym XL^®^ at 8, 10 and 12 hours post-dose [20]. A similar pattern of MPH concentrations was observed with Ritalin LA^®^ (20 mg), which had higher peak MPH concentrations than Concerta^®^ (18 mg) over the first 8 hours post-dose, followed by similar concentrations at 10 hours and lower concentrations at 12 hours post-dose [22]. The biphasic PK profile of Ritalin LA^®^ resulted in a trough in plasma MPH concentration at approximately 5 hours post-dose, which may coincide with a typical lunchtime school break [22]. Following a rapid increase in plasma MPH concentration with a mean peak concentration at 3.7 hours for Ritalin SR^®^ (20 mg; Table 3), plasma MPH concentration declined rapidly compared with Concerta^®^ (18 mg), which had a higher plasma MPH concentration at 8, 10 and 12 hours post-dose [23].

Although AUC_4–12_ and C_max4–12_ were significantly higher for Biphentin^®^ (20 mg) compared with Concerta^®^ (18 mg, p = 0.037 and p = 0.002, respectively; Table 3), plasma MPH concentrations for the two formulations were not significantly different at 8–12 hours post-dose (AUC_8–12_), suggesting the potential for similar efficacy between the two formulations in the evening [25]. For the period covering the school day (AUC_0–8_), plasma MPH concentration for Biphentin^®^ (20 mg) was 128.4% that of Concerta^®^ (18 mg) [25]. The investigators noted that if switching a patient from Concerta^®^ to Biphentin^®^, it may be appropriate to initiate treatment with Biphentin^®^ at a lower daily dose than that of previously received Concerta^®^; however, if switching a patient from Biphentin^®^ to Concerta^®^, the closest marketed dose could be used [25].

As mentioned above, Daytrana^®^ was the only long-acting MPH formulation to reach peak plasma MPH concentration later than Concerta^®^ in head-to-head studies [24]. C_max_, reached at 10 hours post-dose in a single-dose comparison, was greater in children receiving Daytrana^®^ (10 mg/9 hours) compared with those receiving Concerta^®^ (18 mg), and higher in children than adolescents for both MPH formulations (Table 3). This pattern was also observed following multiple fixed doses for 7 days and multiple escalating doses over 28 days [24].

#### Head-to-head PK study of Medikinet^***^®^***^ retard versus Ritalin LA^***^®^***^

In a head-to-head comparison of Medikinet^®^ retard (40 mg) and Ritalin LA^®^ (40 mg), food intake was shown to affect the bioavailability of Medikinet^®^ retard but not that of Ritalin LA^®^[26]. Under fasted conditions, Medikinet^®^ retard showed a steady absorption profile with a single T_max_ in healthy adult volunteers; however, under fed conditions, a biphasic kinetic profile more closely resembling a twice-daily dosing regimen was observed. Food intake also affected overall exposure to MPH (AUC_0–∞_), which was lower in the fasted than in the fed state (Table 3). In contrast, Ritalin LA^®^ (40 mg) had a biphasic kinetic profile under both fasted and fed conditions [26]. Ritalin LA^®^ and Medikinet^®^ retard were bioequivalent in the fasted state but not in the fed state (when a biphasic kinetic profile was observed for both formulations), during which C_max_ for Ritalin LA^®^ was lower compared with that of Medikinet^®^ retard [26]. Haessler and colleagues suggested that, as regular breakfast intake is often challenging in children with ADHD, the unaffected bioavailability with regard to food intake may be a potential advantage of Ritalin LA^®^ over Medikinet^®^ retard [26].

#### Head-to-head PK study of Medikinet***^®^*** retard versus Equasym XL***^®^***

In a head-to-head comparison of Medikinet^®^ retard (20 mg) and Equasym XL^®^ (20 mg), taken as recommended (Medikinet^®^ retard after breakfast, Equasym XL^®^ before breakfast), the two MPH formulations were not bioequivalent in the first 4 hours post-dose (AUC_0–4_) [28]. Medikinet^®^ retard had a slightly higher and slightly earlier peak MPH plasma concentration (mean [standard deviation] 4.83 [1.87] ng/mL at 2.82 [1.00] hours post-dose) compared with Equasym XL^®^ (3.82 [0.96] ng/mL at 3.24 [1.13] hours post-dose; Table 3) [28]. Bioequivalence was demonstrated 4–24 hours post-dose (AUC_4–t_; 90% CI 88.6–103.1), however [28]. While a significant gender x treatment interaction (p = 0.018) for maximum plasma MPH concentration from 4 hours to last observation (C_max4–t_) was noted, no other significant gender effects were observed for other PK parameters [28].

#### Head-to-head PK study of Focalin XR***^®^*** versus Ritalin LA***^®^***

Focalin XR^®^ (20 mg), a long-acting formulation containing pure *d*-MPH, and a 40 mg daily dose of the long-acting racemic MPH formulation, Ritalin LA^®^, were bioequivalent and had very similar plasma MPH concentration profiles over the course of the study [27]. No gender effects on body-weight-adjusted AUC values were reported [27].

#### Effects of MPH formulation on dopamine transport occupancy

Spencer et al. employed positron emission topography to investigate dopamine transporter (DAT) occupancy in the brain 10 hours after dosing with Concerta^®^ (36 mg) and Equasym XL^®^ (40 mg) in 21 healthy adults [21]. Plasma *d*-MPH concentration was also determined 9, 10 and 11 hours post-dose to enable a comparison between peripheral PK and central brain effects. Concerta^®^ resulted in greater plasma *d*-MPH concentrations and greater brain effects (DAT occupancy) at 10 hours compared with a similar daily dose of Equasym XL^®^, suggesting that both peripheral and brain PK profiles can be predicted based on the MPH delivery profile of the long-acting MPH formulation. Plasma concentration was a predictor of DAT occupancy for both MPH formulations, but the correlation between plasma DAT occupancy and *d*-MPH concentration was stronger with Equasym XL^®^ compared with Concerta^®^. While the reasons for this were unclear, the authors noted that this may be associated with a more rapid rate of change in plasma MPH concentration following dosing with Equasym XL^®^, owing to the greater immediate-release component of this MPH formulation compared with Concerta^®^[21].

##### Adverse events

Overall, adverse events associated with long-acting MPH formulations were similar and consistent with the known pharmacological effects of MPH, most commonly including loss of appetite, insomnia, nausea, dizziness, headache and tachycardia [20,23-25,27,28]. Unfortunately, the available data do not permit us to address questions about clinically relevant adverse events specific to different MPH preparations; for example, are the lower peak plasma MPH concentrations observed with long-acting MPH formulations compared with immediate-release formulations associated with lower levels of adverse events, such as appetite loss or increases in blood pressure and pulse rate? Does the absence of a drop in plasma MPH concentration observed towards the end of the 4-hour dosing period for immediate-release MPH result in more consistent appetite suppression? Furthermore, and most pertinent to this review, do the different PK profiles of the long-acting MPH formulations result in different adverse event profiles in certain patients? Reviews of the adverse effects of medication for ADHD, including MPH, can be found elsewhere [29,30].

### Laboratory school studies

Of 34 publications, nine head-to-head comparisons of long-acting MPH formulations were laboratory school studies; eight of which employed Concerta^®^ as a comparator. Two studies each compared Concerta^®^ with Ritalin LA^®^[31,32] and Focalin XR^®^[18,33], while Concerta^®^ was compared with Equasym XL^®^ in four publications derived from one study (the COMACS Study) [34-37]. A single study compared Ritalin LA^®^ with Medikinet^®^ retard [38].

With increasing treatment of school-aged children with stimulant and non-stimulant medications for ADHD, it is important to examine the efficacy and safety of such therapies across the day in an educational setting as well as at home. The laboratory school methodology employs a standardized, regular and repeated cycle of classroom and less-structured activities, representing those encountered in a typical school day, to assess both academic performance and child behaviour in a controlled environment [39]. Regular collection of safety measures also allows the observation of adverse treatment effects in patients with ADHD in a simulated educational setting [39]. Pharmacodynamic data (behaviour and performance) in laboratory school studies are typically collected using the Swanson, Kotkin, Atkins, M-Flynn, Pelham (SKAMP) scale; a questionnaire completed by trained observers at regular intervals [40]. The SKAMP scale comprises six deportment items (interacting with other children, interacting with adults, remaining quiet, staying seated, complying with the teacher’s requests or directions, and following the rules) and seven attention items (getting started on assignments, sticking with tasks, sticking with activities, completing assigned work, performing work accurately, and being neat and tidy while writing or drawing) [40]. An objective measure of academic productivity is provided by a 10-minute written math test administered during the classroom period (consistently used across studies but variably referred to as permanent product or PERMP), from which the number of math test problems attempted (Math-Attempted) and the number correctly answered (Math-Correct) are derived [41]. SKAMP-Attention, SKAMP-Deportment and math tests scores are used as surrogate measures of treatment efficacy. While the main purpose of laboratory school studies is to assess treatment effects in an educational setting, the protocol can be extended into the evening (up to 12 hours post-dose) to assess whether the observed effects extend beyond the traditional school day.

#### Head-to-head laboratory school studies of Concerta***^®^*** versus Equasym XL***^®^***, Ritalin LA***^®^*** and Focalin XR***^®^***

##### Efficacy of long-acting MPH formulations across the day

**Figure 2:**
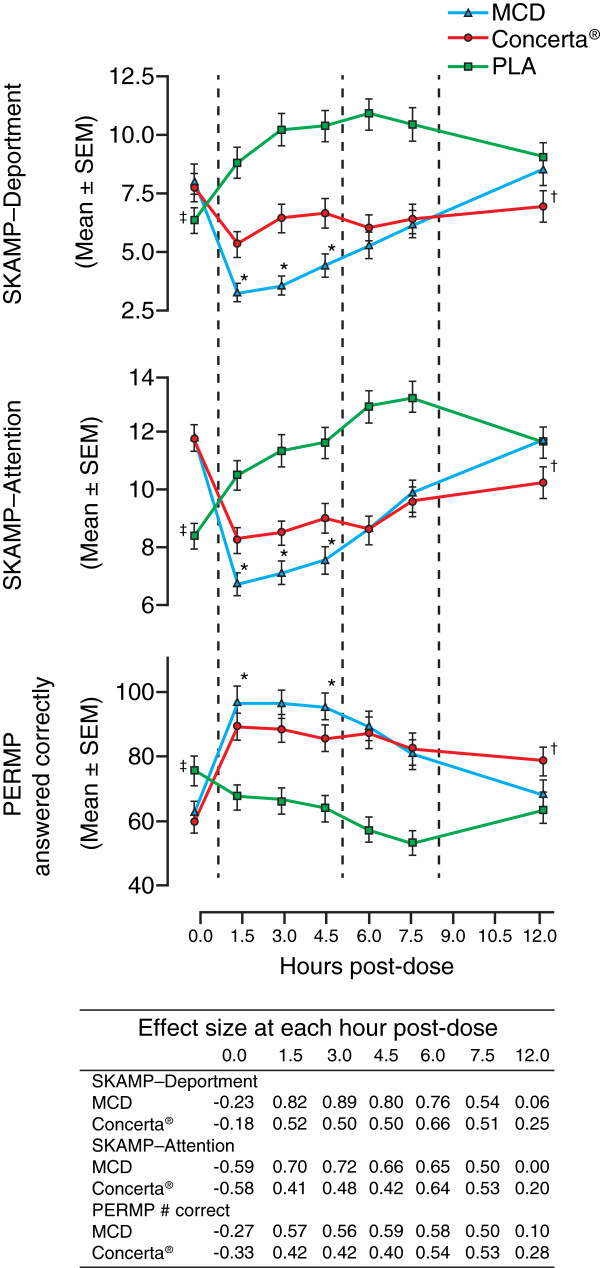
**Comparative efficacy of Concerta^®^and Equasym XL^®^versus placebo over time in a laboratory school study.** Data represent all dose levels combined (Equasym XL^®^ 20, 40, 60 mg; Concerta^®^ 18, 36, 54 mg). Corresponding effect sizes for each timepoint are shown in the table. Originally published in Swanson JM, et al. Pediatrics 2004, 113:e206-e216. Reproduced with permission from Pediatrics, Vol. 113, Page(s) e206–e216, Copyright ©2004 by the AAP. *Equasym XL^®^ was significantly better than Concerta^®^; ^†^Concerta^®^ was significantly better than Equasym XL^®^; ^‡^placebo was significantly better than both Equasym XL^®^ and Concerta^®^. **MCD**, Equasym XL^®^; **PERMP**, permanent product measure of performance; **PLA**, placebo; **SEM**, standard error of the mean; **SKAMP**, Swanson, Kotkin, Atkins, M-Flynn, Pelham rating scale.

Morning: Equasym XL^®^ and Ritalin LA^®^ provided superior symptom control to comparable daily doses of Concerta^®^ in the morning [31,37]. The COMACS Study evaluated differences in the efficacy of bioequivalent doses of Equasym XL^®^ and Concerta^®^ using the laboratory school protocol [37]. Equasym XL^®^ (20, 40, 60 mg) was superior to comparable daily doses of Concerta^®^ (18, 36, 54 mg) for SKAMP-Attention, SKAMP-Deportment and correct math test scores at 1.5–4.5 hours post-dose [37] (Figure 2). Effect sizes for overall, combined dose levels for each formulation, shown in Figure 2, were greatest for Equasym XL^®^ at 3 hours post-dose (SKAMP-Attention 0.72 versus 0.48 for Concerta^®^; SKAMP-Deportment 0.89 versus 0.50 for Concerta^®^). In a post-hoc analysis of the COMACS Study, it was predicted that lower doses of Equasym XL^®^ (20, 40 mg) would provide similar levels of symptom control to 36 and 54 mg doses of Concerta^®^, respectively, in the morning [34]. This hypothesis was based on the similar immediate-release components of the two formulations at the stated, respective, doses. While Equasym XL^®^ 20 mg was associated with a stronger effect and more rapid onset of action than Concerta^®^ (36 mg) at 1.5 hours post-dose, there was no significant overall difference in placebo-adjusted SKAMP scores between the two formulations at 3, 4.5 or 6 hours post-dose and no significant difference in placebo-adjusted SKAMP scores was observed between Equasym XL^®^ 40 mg and Concerta^®^ 54 mg from 1.5 to 6.0 hours post-dose [34].

**Figure 3:**
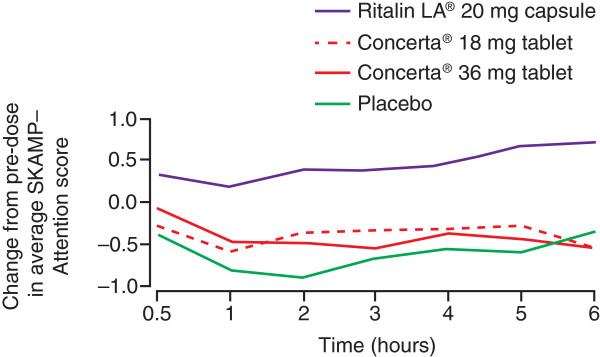
**Comparative efficacy of Concerta**^®^**(18, 36 mg) and Ritalin LA**^®^**(20 mg) versus placebo in a laboratory school study.** Figure shows change from pre-dose baseline in SKAMP-Attention score over time. In the first 4 hours post-dose, Ritalin LA^®^ (20 mg) resulted in significantly greater improvements from baseline than Concerta^®^ (18 mg) in SKAMP-Attention (p = 0.015). Originally published by Springer and Adis Data Information BV/Paediatr Drugs, volume 5, 2003, 545–555, Comparative efficacy of two once daily methylphenidate formulations (Ritalin LA and Concerta) and placebo in children with attention deficit hyperactivity disorder across the school day, Lopez F, et al., Figure 3, ©Adis Data Information BV 2003; with kind permission from Springer Science + Business Media B.V. **SKAMP**, Swanson, Kotkin, Atkins, M-Flynn, Pelham rating scale.

Two laboratory school studies examined the comparative efficacy of Concerta^®^ and Ritalin LA^®^[31,32]. Lopez and colleagues compared Concerta^®^ (18 mg) and Ritalin LA^®^ (20 mg) [31]. In line with observations from PK studies [22], they observed that, in the first 4 hours post-dose, Ritalin LA^®^ (20 mg) resulted in significantly greater improvements from baseline than Concerta^®^ (18 mg) in SKAMP-Attention (p = 0.015) (Figure 3), SKAMP-Deportment (p < 0.001), SKAMP-Combined (p < 0.001) and correct math test scores (p = 0.026) [31]. In contrast, Silva and colleagues demonstrated equivalent efficacy for Ritalin LA^®^ (20 mg) and Concerta^®^ (18 mg) during the first 4 hours (and 8 hours) post-dose for SKAMP-Attention, SKAMP-Deportment and math test scores [32]. It is possible that the findings of Silva and colleagues, however, may be a consequence of including a clinically more heterogeneous study population than the Lopez et al. study. In the study by Silva et al., 64% of subjects had been receiving a stable dose of 40 mg/day MPH prior to enrolment compared with all subjects stabilized to 20 mg/day MPH in the Lopez et al. study. This may have resulted in a suboptimal response in the Silva et al. study [31,32].

Ritalin LA^®^ (20 and 40 mg) also demonstrated superior symptom control compared with the 36 mg dose of Concerta^®^ in the first 4 hours post-dose [31,32]. Lopez and colleagues demonstrated a significantly greater mean change from baseline in SKAMP-Attention (p = 0.043), SKAMP-Deportment (p = 0.004) and SKAMP-Combined (p = 0.003) scores for Ritalin LA^®^ (20 mg) compared with Concerta^®^ (36 mg) in the first 4 hours post-dose [31] (Figure 3). While Silva et al. also demonstrated significant improvements in SKAMP-Attention (p = 0.022) and Math-Correct (p = 0.033) scores for this dose comparison and time period, Ritalin LA^®^ (20 mg) and Concerta^®^ (36 mg) were equivalent in SKAMP-Deportment and Math-Attempted scores [32]. For all efficacy measures, improvements from baseline were significantly greater with Ritalin LA^®^ (40 mg) than with Concerta^®^ 36 mg over the first 4 hours post-dose, evident within 1 hour of dosing and persisting until 8 hours of evaluation [32].

**Figure 4:**
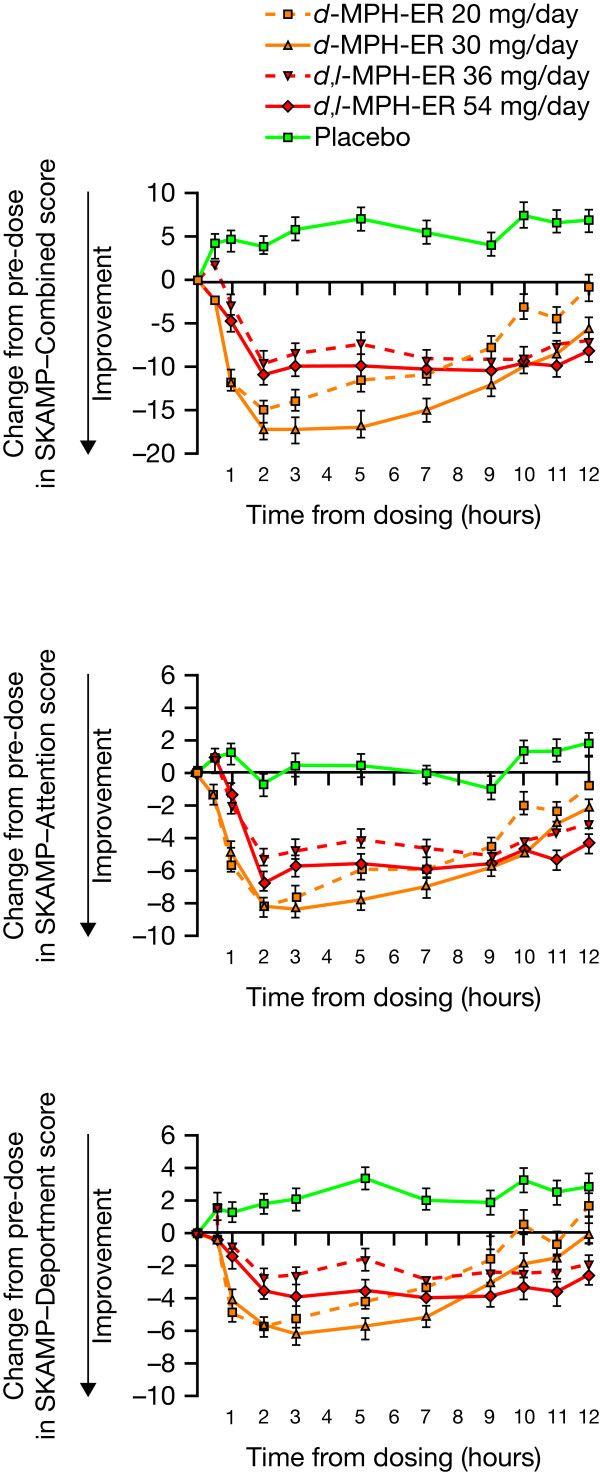
**Comparative efficacy of Concerta**^®^**(*d*,*l*-MPH-ER; 36, 54 mg) and Focalin XR**^®^**(*d*-MPH-ER; 20, 30 mg) versus placebo over time in a laboratory school study. SKAMP**, Swanson, Kotkin, Atkins, M-Flynn, Pelham rating scale. Error bars: standard deviation. Reprinted/Reproduced from Silva R, et al. Psychopharmacol Bull 2008, 41:19–33, with permission from MedWorks Media Global, LLC.

Focalin XR^®^ was superior to higher daily doses of Concerta^®^ (20 versus 36 mg, and 30 versus 54 mg, respectively) at 0.5–6 hours post-dose [18,33]. Two head-to-head laboratory school comparisons of Focalin XR^®^ with higher daily doses of Concerta^®^ (20 versus 36 mg, and 30 versus 54 mg, respectively) demonstrated that Focalin XR^®^ had an earlier onset of efficacy compared with Concerta^®^, with significantly greater improvements from baseline in SKAMP-Combined, SKAMP-Attention, SKAMP-Deportment scores and math test scores with Focalin XR^®^ than with Concerta^®^ at time points between 0.5 and 6 hours post-dose [18,33] (Figure 4). Post-hoc analyses of AUC_0–6_ for SKAMP-Combined scores showed trends nearing statistical significance in favour of Focalin XR^®^ over Concerta^®^ (Focalin XR^®^ 20 mg versus Concerta^®^ 36 mg, p = 0.074; Focalin XR^®^ 30 mg versus Concerta^®^ 54 mg, p = 0.068) [33]. Significantly greater improvements from baseline with Focalin XR^®^ compared with Concerta^®^ were also observed in Math-Attempted and Math-Correct scores at 3 hours (20 versus 36 mg) and 4–5 hours (30 versus 54 mg) post-dose [18,33].

Afternoon and evening: While the COMACS Study demonstrated the superior efficacy of Equasym XL^®^ compared with Concerta^®^ in the morning, similar efficacy of comparable daily doses of the two MPH formulations was observed at 6.0–7.5 hours post-dose, and Concerta^®^ demonstrated superiority over comparable doses of Equasym XL^®^ at 12 hours post-dose [37] (Figure 2). In cross-dose comparisons, lower doses of Concerta^®^ (18 and 36 mg) provided equivalent symptom control to higher doses of Equasym XL^®^ (40 and 60 mg, respectively) at 7.5 hours and 12 hours post-dose [34]. In a reversed-dose comparison, a lower dose of Equasym XL^®^ (20 mg) was comparable at 7.5 hours post-dose with a higher dose of Concerta^®^ (36 mg) but Concerta^®^ (36 mg) was superior to Equasym XL^®^ (20 mg) at 12 hours post-dose [34]. However, when Equasym XL^®^ (40 mg) was compared with Concerta^®^ (54 mg) in a similar cross-dose comparison, Concerta^®^ was superior to Equasym XL^®^ at both 7.5 and 12 hours post-dose [34].

Over the 8-hour classroom period employed by Lopez et al., Ritalin LA^®^ (20 mg) resulted in a significantly greater mean change from baseline (AUC_0–8_) compared with Concerta^®^ (18 mg) in SKAMP-Combined (p = 0.010) and SKAMP-Deportment (p = 0.018) scores and demonstrated trends towards significance in SKAMP-Attention (p = 0.074) [31]. Ritalin LA^®^ (20 mg) also demonstrated trends towards superiority over a higher daily dose of Concerta^®^ (36 mg) in SKAMP-Combined (p = 0.061) and SKAMP-Deportment (p = 0.078) scores over the 8-hour assessment period [31]. Silva and colleagues aimed to replicate and extend the findings of Lopez et al. using a similar study design but a longer, 12-hour classroom protocol. However, in contrast with Lopez et al., Silva and colleagues demonstrated comparable efficacy of Ritalin LA^®^ (20 mg) and Concerta^®^ (18 mg) over the first 8 hours post-dose (AUC_0–8_), possibly, as stated earlier, owing to a clinically more heterogeneous study population [32]. Using the extended 12-hour classroom protocol, Silva et al. observed significantly greater changes from baseline at 8–12 hours post-dose (AUC_8–12_) in SKAMP-Combined and SKAMP-Deportment scores with Concerta^®^ 18 and 36 mg compared with Ritalin LA^®^ 20 mg (but not 40 mg), and significantly more correct math test responses with Concerta^®^ 36 mg than with Ritalin LA^®^ 20 mg (p = 0.046) were observed [32].

While post-hoc analyses of AUC_0–6_ for SKAMP-Combined scores showed trends, nearing statistical significance, favouring Focalin XR^®^ (20 and 30 mg) over higher daily doses of Concerta^®^ (36 and 54 mg), differences between the two MPH formulations from 6 to 12 hours post-dose (AUC_6–12_) failed to reach significance (20 versus 36 mg, p = 0.244; 30 versus 54 mg, p = 0.594) [33]. Although Concerta^®^ and Focalin XR^®^ demonstrated similar efficacy at 7–9 hours post-dose [18], Concerta^®^ demonstrated significantly greater improvements in SKAMP-Combined, SKAMP-Attention and SKAMP-Deportment scores at 10–12 hours post-dose compared with lower daily doses of Focalin XR^®^ (36 versus 20 mg, and 54 versus 30 mg, respectively) [18,33].

Both laboratory school studies comparing Concerta^®^ with Focalin XR^®^ employed the Conners’ Parent Rating Scale (CPRS) to obtain additional parental ratings of their child’s behaviour during the previous week. Muniz and colleagues demonstrated that, while Focalin XR^®^ (20 mg) had a significantly greater effect on parent-rated symptom control than Concerta^®^ (36 mg), no significant differences between the change in CPRS scores for Focalin XR^®^ 30 mg and Concerta^®^ 54 mg were observed [33]. In contrast, Silva et al. found no significant difference for change from baseline in CPRS scores, between formulations for the lower dose comparison (Focalin XR^®^ 20 mg versus Concerta^®^ 36 mg) and found Concerta^®^ (54 mg) to be superior to Focalin XR^®^ (30 mg); however, no explanation is provided for this disparity between study findings [18].

#### Head-to-head laboratory school study of Ritalin LA***^®^*** versus Medikinet***^®^*** retard

**Figure 5:**
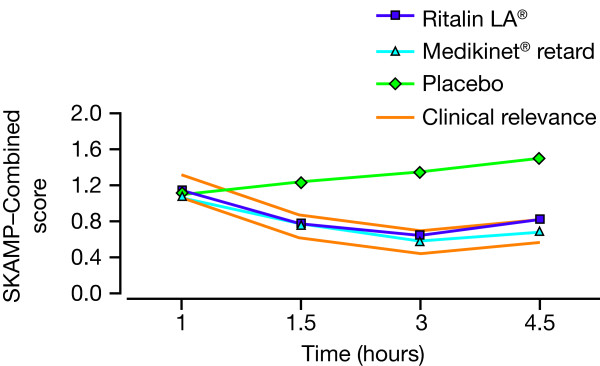
**Comparative efficacy of Ritalin LA^®^(20 mg) and Medikinet^®^retard (20 mg) versus placebo in a laboratory school study.** Figure shows SKAMP-Combined score over the first 4.5 hours post-dose. 'Clinical relevance’ lines outline the predefined non-inferiority margin. **SKAMP**, Swanson, Kotkin, Atkins, M-Flynn, Pelham rating scale. Originally published in Schulz E et al. J Child Adolesc Psychopharmacol 2010, 20:377–385. Reproduced with permission. The publisher for this copyrighted material is MaryAnn Liebert, Inc. publishers.

One laboratory school study compared Ritalin LA^®^ (20 mg) with Medikinet^®^ retard (20 mg) using a 7.5 hour laboratory school protocol and found no clinically relevant differences between the two MPH formulations [38] (Figure 5). Both treatment groups demonstrated comparable improvements in SKAMP-Combined score and math test scores until peak efficacy was reached at 3 hours post-dose [38]. Change from screening visit in Nisonger Child Behaviour Rating Form score (a parent-rated assessment of child and adolescent behaviour) demonstrated that both Ritalin LA^®^ and Medikinet^®^ retard improved disruptive behaviours [38].

#### Effect of symptom severity on treatment choice

A secondary analysis of the COMACS Study using growth mixture modelling analysis (a statistical technique to identify subgroups within a population with different trajectories of change over time) of total SKAMP scores investigated the effect of symptom severity on MPH response to Equasym XL^®^ and Concerta^®^[36]. Results suggested heterogeneity in pharmacodynamic response to MPH by children with ADHD that is dependent on both symptom severity and MPH formulation. Sonuga-Barke and colleagues found that, with increasing severity of ADHD symptoms, the larger immediate-release bolus of Equasym XL^®^ provided greater symptom control in the morning compared with near-equal and bioequivalent doses of Concerta^®^ (effect size 0.59 and 0.77 at 1.5 and 3 hours post-dose, respectively, in children with high symptom severity; effect size 0.58, 0.60 and 0.54 at 1.5, 3 and 4.5 hours post-dose, respectively, in children with intermediate symptom severity) [36]. The efficacy of Concerta^®^ was unaffected by symptom severity, thus the difference between the pharmacodynamic profiles of the two MPH formulations became more evident as symptom severity increased. The predicted superiority of Concerta^®^ at 12 hours post-dose was observed only in children with intermediate symptom severity (effect size 0.37). It must be noted that these findings may not translate directly into clinical practice, however, as the subgroups of symptom severity were identified according to performance in the placebo condition in the laboratory classroom setting and not on the basis of parent or teacher ratings in the home or school environment [36].

#### Effect of gender on response to MPH

A significant effect of gender on response to MPH was observed in a further secondary analysis of the COMACS Study [35]. This was independent of MPH formulation (Concerta^®^ or Equasym XL^®^), however. Females demonstrated a superior response to MPH, measured using SKAMP-Combined scores (controlled for placebo and baseline scores, and for the presence of comorbid anxiety) when compared with males at 1.5 and 3 hours post-dose, an equivalent response to males between 4.5 and 6 hours post-dose and a greater decline in response compared with males between 7.5 and 12 hours post-dose [35]. Analyses using PERMP scores confirmed this faster decline in response to MPH in females compared with males [35]. The response of female patients to MPH may, therefore, require additional assessments later in the day to determine the optimal dose of MPH [35]. Unfortunately, as most studies include only small numbers of females, the power of other head-to-head studies to investigate the effect of gender in MPH response is limited. While a significant gender by treatment interaction (p = 0.018) for maximum plasma MPH concentration from 4 hours to last observation (C_max4–t_) was noted in a head-to-head PK study of Equasym XL^®^ and Medikinet^®^ retard performed in healthy adults, no other significant gender effects were observed [28]. In addition, no gender effects were noted in a head-to-head PK study of Focalin XR^®^ and Ritalin LA^®^[27].

#### General observations and adverse events from laboratory school studies

An important general observation from the laboratory school studies comparing Equasym XL^®^ and Concerta^®^ is that superiority at any point in time was achieved by the formulation with the highest expected plasma MPH concentration (predicted from PK data available) [37,42]. Despite dose selection based on clinical titration, the size of the drug effect obtained in the early morning appears to be directly related to the absolute dose delivered by the immediate-release MPH bolus of each formulation [37,42]. The duration of action of clinical effects is also in line with what would be predicted from PK data. Adverse events for all of the oral long-acting MPH formulations were generally mild to moderate in severity and commonly included abdominal pain, headache and decreased appetite [18,31-33,37,38]. While adverse events were generally similar between different formulations, in one study treatment-related abdominal pain and anorexia were more frequent in subjects receiving Medikinet^®^ retard (5/147; 3.4% and 6/147; 4.1%, respectively) than those receiving Ritalin LA^®^ (1/147; 0.7% and 3/147; 2.0%, respectively) [38]. This difference in frequency was not assessed for significance and the investigators concluded that there were no relevant differences between Medikinet^®^ retard and Ritalin LA^®^ regarding the profile, frequency or intensity of adverse events [38].

### Head-to-head RCTs of long-acting MPH formulations

To date, only two head-to-head non-laboratory school RCTs have been performed, both of which included Concerta^®^ as a comparator.

#### Concerta***^®^*** versus Daytrana***^®^***

In a Phase III, double-blind, double-dummy, parallel-group, placebo-controlled, naturalistic home and school trial, children (n = 282) were randomized to receive either Concerta^®^, Daytrana^®^ or placebo [43]. Following a 5-week dose optimization period, children who reached an acceptable level of efficacy and tolerability entered a 2-week dose-maintenance phase during which assessment of treatment efficacy and safety were performed at the end of each week. Blood samples were collected from participants at 7.5, 9 and 10.5 hours post-dose during one of the last three study visits for determination of plasma MPH concentration. By the end of the dose optimization period, the majority of children were receiving Concerta^®^ at a dose of 36 mg (32.4%) or 54 mg (44.1%) or Daytrana^®^ at 20 mg/9 hours (34.2%) or 30 mg/9 hours (36.8%). Results at study endpoint revealed no significant differences between Concerta^®^ and Daytrana^®^ for: mean change from baseline in ADHD Rating Scale-version IV (ADHD-RS-IV) scores (the primary efficacy measure of the study); mean change from baseline in Conner’s Teacher Rating Scale-Revised (CTRS-R) total score; or mean change from baseline in CPRS-R total score in the morning or the afternoon. Furthermore, the majority of children receiving Daytrana^®^ (71.9%; n = 69) and Concerta^®^ (66.3%; n = 59) were rated as improved using the Clinical Global Impressions–Improvement (CGI–I) scale at study endpoint, while 69.8% (n = 67) and 60.7% (n = 54), respectively, were rated as improved using the Parent Global Assessment (PGA) scale.

Higher plasma concentrations of *d*-MPH and *l*-MPH were observed after 9 hours of treatment with Daytrana^®^ compared with 9 hours post-dosing with Concerta^®^, indicating that greater systemic exposure to MPH is observed in the latter part of the day with Daytrana^®^.

#### Concerta***^®^*** versus Medikinet***^®^*** retard

A randomized, double-blind, cross-over study design was used to investigate the efficacy of Concerta^®^ and Medikinet^®^ retard with equivalent daily doses (but different immediate-release components) and different daily doses (but similar immediate-release components) in the school and home environment [42]. Efficacy was rated by teachers using the German version of the SKAMP scale (SKAMP-D), while both teachers and parents rated ADHD symptoms using the Day Profile of ADHD Symptoms (DAYAS) [42].

Medikinet^®^ retard with a higher immediate-release component and similar daily dose to Concerta^®^ was superior to Concerta^®^ (20 versus 18 mg and 30 versus 36 mg, respectively) in the first 3 hours of school and 4–6 hours into the school day, as assessed using SKAMP-D [42]. Medikinet^®^ retard with a similar immediate-release component to Concerta^®^ in the morning but with a lower daily dose was non-inferior to Concerta^®^ (10 versus 18 mg and 20 versus 36 mg, respectively) in the first 3 hours and 4–6 hours of the school day (SKAMP-D) [42]. No evidence for the superiority of Concerta^®^ over Medikinet^®^ retard with equivalent daily doses in the late afternoon and evening was observed using DAYAS teacher or parent ratings [42].

##### Adverse events

No significant difference in the frequency of adverse events was observed between Concerta^®^ and Daytrana^®^; the majority of adverse events were mild to moderate in severity [43]. Application-site reactions were noted as being among adverse events resulting in study discontinuation for patients receiving Daytrana^®^. While mild erythema was common, 77% of subjects reported either no or minimal evidence of irritation. A higher incidence of tic disorders was observed in patients receiving Daytrana^®^ (n = 7, 7.1%; nine events) compared with Concerta^®^ (n = 1, 1.1%; one event); however, this was deemed unlikely to reflect a greater risk of tics associated with Daytrana^®^[43-45]. The most frequent adverse events noted in RCTs were headache, abdominal pain, decreased appetite, nausea, vomiting and insomnia [42,43]. In addition, there was no evidence of differences in overall tolerance (assessed by the investigator, parents and teachers) between Concerta^®^ and Medikinet^®^ retard [42].

### Switching and observational studies

Of 34 publications included in the review, three publications derived from two open-label switching studies [46-48] and three publications derived from one observational study [49-51] were identified. All studies included Concerta^®^ as a comparator.

#### Switching studies

In one switching study, children aged 6–12 years with ADHD on a stable dose of oral long-acting MPH (Concerta^®^, Equasym XL^®^ or Ritalin LA^®^), not exceeding 54 mg/day, underwent abrupt switching to Daytrana^®^ using a pre-defined dose-transition schedule. Titration was based on changes in ADHD-RS-IV score and Clinical Global Impressions–Severity (CGI–S) scale score. Measures of ADHD symptoms and quality of life were obtained using ADHD-RS-IV, CPRS-R, CGI–S, CGI–I, PGA and the ADHD Impact Module-Child (AIM-C) [46,47].

Abrupt conversion from oral long-acting MPH formulations to an optimum dose of Daytrana^®^ using a dose-transition schedule was not associated with deterioration of symptom control [46,47]. After 1 week of treatment with Daytrana^®^, the majority of children (78%) had a CGI–I score indicating improvement or no change and mean ADHD-RS-IV total score was similar to that at baseline, indicating little change in ADHD symptoms when subjects switched to Daytrana^®^ from oral long-acting MPH formulations [46]. After 4 weeks of treatment with Daytrana^®^, 96% of children had CGI–I scores rated as 'improvement or no change’ relative to baseline. Furthermore, a significant improvement from baseline was observed in ADHD-RS-IV mean total score (p < 0.0001). Forty-two percent of children received dose-optimization adjustments during the study, most of which were dose increases (38% of subjects) [46]. Optimal nominal doses of Daytrana^®^, which ranged from 10 mg/9 hours (n = 23) to 30 mg/9 hours (n = 59) were below the nominal doses of oral long-acting MPH from which subjects were switched. Clinicians could consider initiating treatment with Daytrana^®^ at a smaller patch size than the oral dose received previously. However, treatment for each patient should be optimized on an individual basis [46,47].

Improvements from baseline in behaviour (PGA, CPRS-R), health-related quality of life (HRQoL; AIM-C) for both the child and the family, compliance and economic impact (number of missed school days; hours of tutoring, nursing, home healthcare and use of other services required; number of work days missed by parent/caregiver) were also observed after treatment for 4 weeks with Daytrana^®^[47]. Differences between subgroups were noted: greater improvements from baseline to endpoint in child HRQoL were reported for: children switching from Ritalin LA^®^; children aged 6–9 years; and females rather than males [47]. In addition, greater improvement in the AIM-C School/Missed-Doses Worry Scale was observed in subjects who switched to Daytrana^®^ from Equasym XL^®^ than in those who switched from Concerta^®^ or Ritalin LA^®^[47]. For all prior-treatment groups, caregiver satisfaction with Daytrana^®^ treatment was high and 82.6% of caregivers reported improvements in their child’s social interactions since switching to Daytrana^®^[47]. According to the authors, the apparent superiority of Daytrana^®^ observed during the study, however, is more likely to be due to careful titration and clinical monitoring for the study duration rather than to the product itself [46,47].

In a second switching study, 308 children with ADHD who were either untreated or currently receiving treatment with MPH (immediate-release MPH, extended-release MPH excluding Concerta^®^, or Concerta^®^) were switched to Equasym XL^®^ for 3 weeks [48]. Children currently receiving MPH started Equasym XL^®^ at a dosage based on clinical judgement and the patient’s current MPH dose, while previously untreated children started Equasym XL^®^ treatment at a dose of 20 mg. Equasym XL^®^ was titrated for all patients on a weekly basis according to clinical judgement. Measures of ADHD symptom control and therapeutic response were obtained at weeks 1, 2 and 3 using CGI–I and the CGI–Efficacy Index and compared with baseline scores. Of those who were previously receiving ADHD medication (181; 59%), most switched from Concerta^®^ (41.4%) or immediate-release MPH (36.5%) with only 9% switching from a long-acting MPH other than Concerta^®^. Overall, 60.6% of children switching from a previous MPH formulation were responders to Equasym XL^®^ (CGI–I score of 1 [very much improved] or 2 [much improved]) at week 3 and approximately half (55%) of children switching from Concerta^®^ demonstrated improvement from baseline in CGI–I at week 3. The majority of children (63%) switching from a previous MPH treatment also demonstrated a moderate or marked therapeutic effect with either no or minimal side-effects, as measured using the CGI–Efficacy Index. The authors suggested that improvements observed in patients previously receiving MPH may be due to treatment optimization and differences in PK between the two MPH formulations.

##### Adverse events

Consistent with commonly reported adverse events associated with MPH, 59.6% of caregivers agreed that Daytrana^®^ decreased their child’s appetite and 34.8% agreed that treatment with Daytrana^®^ made it more difficult for their child to fall asleep at night [47]. Such late-day side effects may be attenuated by early removal of the patch [44]. Adverse events with Daytrana^®^ and Equasym XL^®^ were mostly mild to moderate in severity and most commonly included headache, decreased appetite, insomnia and abdominal pain [48]. Despite a transdermal route of administration, Daytrana^®^ was associated with those adverse events typically observed with oral long-acting MPH, with the added issue of application-site reactions that included normal appearance at the patch site with moderate itching, as well as erythema with severe itching. However, most subjects reported no or mild discomfort [46,47]. One subject reported two serious adverse events (acute depression and suicide attempt) while receiving 30 mg Daytrana^®^ for 16 days that were considered possibly related to treatment.

#### Observational study

The OBSEER study was a non-interventional, non-controlled, observational study. Patients with ADHD intended for treatment with Equasym XL^®^, either previously treatment-naive, receiving treatment with other MPH formulations (immediate-release or long-acting, most commonly immediate-release Medikinet^®^ and Medikinet^®^ retard, respectively), receiving a different pharmacological therapy or receiving non-pharmacological therapy were observed for 6–12 weeks in routine care. As this was a non-interventional study, treatment optimization was not part of the study remit and MPH dose adjustments were at the discretion of the treating physician. Measures of ADHD symptoms and quality of life were obtained using the CGI–S scale, the German ADHD symptom checklist (Fremdbeurteilungsbogen für Aufmerksamkeitsdefizit–Hyperaktivitätsstörung [FBB-ADHD]; rated by teachers and parents), DAYAS (rated by teachers and parents) and a HRQoL questionnaire (Kinder Lebensqualitätsfragebogen [KINDL]) [49]. Despite most children (69.8%) previously receiving MPH medication, improvement in ADHD symptoms was observed following the switch to Equasym XL^®^[49]. The largest reduction in clinician-rated ADHD symptoms (CGI–S) was observed in the treatment-naive subgroup (Cohen’s *d* = 1.73); however, a reduction in CGI–S was also observed in patients previously receiving a long-acting MPH formulation (Cohen’s *d* = 0.76) [50].

The reduction in parent-rated ADHD symptoms (Cohen’s *d* = 0.79; FBB-ADHD) was larger than the reduction in teacher-rated ADHD symptoms (Cohen’s *d* = 0.41; FBB-ADHD) [50]. For parent-rated DAYAS scores, the largest reduction in ADHD and oppositional defiant disorder symptoms in patients previously receiving a long-acting MPH formulation was observed in the early afternoon (Cohen’s *d* = 0.63), with smaller but still substantial improvements observed in the morning before school, late afternoon and evening (Cohen’s *d* range 0.44–0.49). No significant difference was observed between prior treatment subgroups in the evening [50]. Less improvement was noted in teacher-rated DAYAS scores for the first 2–3 hours and second 2–3 hours of the school morning (Cohen’s *d* = 0.14 and 0.39, respectively) compared with parent-rated DAYAS scores for the morning before school (Cohen’s *d* = 0.49), afternoon until 4 pm (Cohen’s *d* = 0.63), late afternoon until 7 pm (Cohen’s *d* = 0.45) and evening (Cohen’s *d* = 0.44) [50]. While parents and physicians were not blinded to study treatment or dose, teachers were not formally notified of the change in treatment. The lower effect sizes in the teacher ratings may be a more accurate representation of treatment effect, therefore, as they were not influenced by expectation and dissatisfaction with prior treatment [49].

Improvement following the initiation of treatment with Equasym XL^®^ was also observed in mean KINDL score and KINDL scales for self-esteem, friends and school (parent and patient ratings) and family (patient ratings). The largest effect size in patients formerly receiving long-acting MPH in parent-rated quality of life was on the KINDL *friends* scale (Cohen’s *d* = 0.42), while the largest effect size noted for patient-rated quality of life was on the KINDL *family* scale (Cohen’s *d* = 0.37) [50,51]. Overall, adherence during Equasym XL^®^ treatment was frequently rated as superior to adherence during prior treatment; however, 12.8% of patients previously receiving long-acting MPH had better adherence to prior treatment, compared with 8.0% for all treatments overall [51].

##### Adverse events

The most frequent adverse events recorded during the OBSEER study were psychiatric disorders (19.8% of all patients), metabolism and nutrition disorders (2.4%), and gastrointestinal disorders (2.2%), with tics being the most frequent single adverse event recorded (106 events in 100/822 [12.2%] patients). While the frequency of tics was high, the authors noted that conclusions regarding the emergence of treatment-related tics were limited as patients with pre-existing tics were not excluded from the study and emergent tics were not differentiated from those pre-existing. Serious adverse events (n = 38) were recorded for 21/822 (2.5%) patients, which the investigators noted was high compared with previous studies. The investigators proposed that this may be due to various factors, including the long duration of observation in the OBSEER study, a lack of data regarding whether the adverse events were present under the previous medication, missing data for 10.7% of patients and possible incorrect categorization of adverse event seriousness by the investigators.

### Meta-analyses and systematic reviews

Eight systematic reviews and meta-analyses addressed long-acting ADHD medications, including, but not limited to, MPH for the treatment of ADHD. An overview of the main conclusions of these reviews is presented.

Consideration of the onset and duration of efficacy of long-acting MPH formulations in the context of the patient’s individual needs when selecting an appropriate formulation was highlighted [19,52,53]. The pattern of efficacy generally follows that predicted by the PK profile of the MPH formulation [53]. As such, efficacy offset varies between long-acting MPH formulations, although whether this is clinically perceptible outside the research setting is unknown [19,53]. Greater efficacy during the first 8 hours post-dose compared with later in the day may be beneficial for parents/caregivers during the pre-school period (getting the child ready for school and travelling to school) and during the school day; however, some families may prefer greater symptom control in the evenings to improve concentration for homework completion, or for greater behavioural control in social/familial interactions [19,53]. Selection of the optimal MPH formulation may be influenced by the individual’s sensitivity to appetite problems and insomnia; therefore, MPH formulations with shorter duration of action may be more appropriate over longer-acting formulations to reduce interference with dinnertime and sleep [52,54].

With regard to administration of medication, Equasym XL^®^, Medkinet^®^ retard and Ritalin LA^®^ capsules can be opened and sprinkled on food, which may have a compliance benefit over Concerta^®^ in patients who have difficulty swallowing. However, surreptitious administration of medication by parents/caregivers should not be undertaken as this may result in a lack of trust between the patient and parent/caregiver [52].

The importance of head-to-head studies for the direct comparison of the efficacy of different medications was highlighted but the lack of uniformity in study design parameters used to assess medication efficacy, particularly for studies assessing long-acting stimulants was noted as a significant limitation with current studies [55-57].

In meta-analyses examining treatment efficacy in adults with ADHD, Peterson et al. found that short-acting stimulants were more effective than long-acting stimulants in the treatment of adults with ADHD [58]. While an initial analysis by Faraone et al. supported this finding, no significant difference between the effect sizes for long- and short-acting stimulants in the treatment of adults with ADHD was detected after study confounders and publication bias were accounted for [57]. Furthermore, differences in study methodology (inclusion/exclusion criteria, outcome measures analysed and literature search timing) were also suggested to contribute to the contradiction in findings from the two meta-analyses [57].

## Conclusions

The objective of this review was to bring together the evidence available from head-to-head studies of long-acting MPH formulations and to increase understanding of their basic properties, discuss similarities and differences, and provide information that can guide treatment selection.

In addition to supporting the conclusions of existing meta-analyses and systematic reviews on long-acting MPH formulations, our review of head-to-head studies reinforces the finding that, at a group level, the pattern of efficacy across the day generally follows that predicted by the PK profile of the formulation. The timecourse of both plasma MPH concentration and central brain effects (DAT occupancy) may be predicted based on the MPH delivery profile of a long-acting formulation [21]. It must be noted, however, that there is significant variability in PK profiles across the day at an individual level and that, as a consequence of this, the individual response to any given product and dosing strategy may vary substantially. The clinical consequences of this variability are that no one treatment is superior for all patients and that individualized treatment optimization is an important clinical task. To make the best use of the various long-acting MPH preparations clinicians need to understand the similarities and differences between them and how to harness these to achieve the best results for their patients.

For patients achieving significant but suboptimal effects with a long-acting MPH medication, switching to another MPH formulation should be considered. This advice relates to situations in which there has been at least a partial response to MPH (e.g., adequate symptom coverage for a certain period of the day) and the clinician is trying to fine tune and optimize treatment. Such an approach may prove beneficial and can often be undertaken without loss of symptom control during the period of transition from one formulation to another. As noted previously, responses to formulations may vary between individuals and the time–action profile of the medication should be considered during switching and tailored to the patient’s needs. The availability of the different long-acting MPH formulations varies across the world, and even within continents, and clearly impacts on the options available to the clinician. At the present time the greatest range is available to patients in the USA (Table 1). In cases where a lower daily dose of MPH is preferred, data have shown that children and adolescents could be treated with a lower daily dose of Medikinet^®^ retard and Daytrana^®^ than Concerta^®^ without clinically relevant deterioration in symptom control during school time [42,47,50]. However, it is also acceptable to increase the daily dose of MPH in order to achieve optimal symptom control [52] and indeed higher daily doses should not necessarily be seen as negative [59]. Clinicians are often uncertain about correct dosing when switching patients from immediate- or extended-release MPH to a long-acting MPH formulation with an immediate-release component of <50% (Concerta^®^ in particular). As a consequence, many patients receive suboptimal treatment. It is usually appropriate to use the immediate-release component of each formulation as the reference and try to adjust for this when switching between MPH formulations. A limitation of current studies is that they have mostly focused on the total daily dose rather than equivalent immediate-release components. Data from head-to-head studies of long-acting MPH formulations suggest that, across formulations, equivalent immediate-release components provide similar symptom control in the morning and this would be our clinical suggestion. As an example, if one wants to switch a patient from 20 mg of Medikinet^®^ retard (50:50 immediate- and extended-release) to Concerta^®^ (22:78 immediate- and extended-release) to try to alleviate breakthrough symptoms at the end of the school day, then 45 mg of Concerta^®^ would give the equivalent immediate-release dose. Although the supporting data are not reviewed here, when there is little clinical response to MPH at the end of a careful titration, switching from MPH to another stimulant or a non-stimulant medication is likely to be the most beneficial for such patients who are poor responders to MPH.

Duration of required symptom control may vary between individuals. Although it has been argued that an ascending PK profile is required to combat acute tolerance [60] it is likely that for some individuals, reaching a peak plasma MPH concentration at a minimum of 6–8 hours post-dose, as observed for Concerta^®^ and Daytrana^®^, may be quite late in the day as the school/work morning is over and symptom control may not be optimal when required. In such cases clinicians may favour one of the 8-hour formulations. However, there is also evidence that for many patients, ADHD symptoms continue into the late afternoon and evening [61]. Where this is the case, extending symptom control beyond 8 hours has the potential to benefit many, if not most, patients with ADHD. This may be particularly important for adults and adolescents, who are often required to maintain high levels of functioning over these periods. The various long-acting MPH formulations allow for flexibility in duration of symptom control, which can be tailored to the individual patient’s needs. Twelve-hour symptom coverage can be obtained using a formulation such as Concerta^®^ on its own, or by combining one of the 8-hour preparations with an additional immediate-release dose at around 4 pm. Female patients have been shown to have a faster decline in response to MPH compared with males [35] and may require closer assessments of their afternoon symptom control to determine optimal MPH dose.

Flexibility in how the medication can be taken may be of particular benefit for some patients. For example, the ability to open capsules and sprinkle the medication on food may be of benefit for patients who have difficulty taking tablets and offers an advantage for pre-school children who are not yet able to swallow pills. If regularly eating breakfast is a challenge for the patient, a formulation for which bioavailability is not affected by food intake may be preferred. In such cases, Ritalin LA^®^ may have a potential advantage over Medikinet^®^ retard [26]. Alternatively, a transdermal rather than an oral mode of action may be preferred by some patients. While the side-effect profiles are similar between different long-acting MPH formulations, Daytrana^®^ may be associated with application-site reactions.

This review has highlighted several unmet needs. While we recognize that the evidence to date suggests that the profile of clinical action across the day can generally be predicted from the PK profile of a particular preparation, the direct evidence to support this proposition is almost all from head-to-head studies that compared Concerta^®^ with one other long-acting MPH formulation. The laboratory school protocol seems to be the ideal methodology for addressing outstanding questions; however, at the time of writing, only one laboratory school study had been performed in which Concerta^®^ was not the comparator. This study compared equivalent daily doses of Ritalin LA^®^ and Medikinet^®^ retard, both of which have a 50/50 immediate-release/extended-release delivery profile, and showed no clinically relevant differences between the two formulations [38]. We therefore believe that more head-to-head laboratory school studies of alternative combinations of long-acting formulations are required to provide evidence-based guidance on treatment selection and guide the development of clinical guidelines, and to inform the decisions of regulators and those making decisions about reimbursement and ultimately the decisions made in day-to-day clinical practice. In particular, further studies comparing the efficacy of formulations containing racemic *d,l*-MPH with *d*-MPH (Focalin XR^®^) would be of particular interest. Pragmatic head-to-head studies looking at dose optimization across the day in the short- and long-term, and longer-term comparative studies to assess efficacy and safety over time, are also required as well as laboratory school studies comparing MPH with other ADHD medications.

In addition to these studies, which would be applicable to all ages, further studies using an age-appropriate laboratory school-style protocol in adults are needed to assess real-life medication effects across the day. More studies of the effects of long-acting MPH formulations in pre-school children, both within age-appropriate laboratory school settings and assessing their impact in more naturalistic settings on developmental and academic outcomes would also be of interest. Further research into the effect of comorbidities and symptom severity as modifiers of treatment response with the various long-acting MPH formulations available is also necessary. Compliance and adherence may differ between different long-acting medications; however, it is unknown whether this is a true reflection of medication adherence or an effect of study involvement. More studies investigating the impact of the different formulations and related dosing strategies on adherence could throw light on these questions.

This review has a number of potential limitations. While two extensive databases were searched to identify relevant articles for inclusion in the review, the authors are aware of studies that were not retrieved by the search terms. These include an open-label study of 447 children and adolescents switching from immediate-release MPH, extended-release MPH, or no treatment, to Medikinet^®^ retard [62]. Significant improvements from baseline at 4–6 weeks were observed in ADHD symptom severity, as evaluated by physicians and parents, in patients switching from Concerta^®^ (n = 64) but not in those switching from Ritalin SR^®^/Ritalin LA^®^ (n = 26). The superior efficacy of Medikinet^®^ retard compared with Concerta^®^ is probably due to the larger immediate-release bolus from Medikinet^®^ retard (50%, versus 22% for Concerta^®^), while the lack of significant improvement with Medikinet^®^ retard compared with Ritalin SR^®^/Ritalin LA^®^ may be explained by the motivation of patients to participate in the study, and not necessarily because the prior therapy was suboptimal.

The diversity of head-to-head studies of long-acting MPH formulations and their reported outcomes creates a challenge when drawing general conclusions and providing clinical recommendations. Emerging studies provide important data on the comparative efficacy of formulations available, but further studies are necessary to provide more evidence-based guidance for clinical practice. Effect sizes have been cited in the review where appropriate; however, heterogeneity in study design and reported outcomes precluded the undertaking of meta-analysis.

Despite these limitations there are several clear messages for clinicians using long-acting MPH preparations to treat patients with ADHD. Different patients have both different treatment needs and responses to MPH. There is now clear evidence that, in order to optimize the treatment of ADHD symptoms, a tailored approach to treatment is required. This involves both an initial titration onto medication and a continued follow up, with careful adjustments in dose and often in MPH formulation. It is important to track symptoms and response across the day. One possible tool for this is the Dundee Difficult Times of the Day Scale (D Coghill, personal communication, available from [63]). It is clear from this review that no one long-acting MPH preparation is clearly superior to another. However, even though some of the formulations are very similar to each other, each has its own particular profile and there are differences with respect to mode of delivery, PK/pharmacodynamic profile, dosing, duration of action, interaction with food and adverse effects. In addition to carefully collecting information about ADHD symptoms and the way that they change across the day, clinicians need to be aware of the, often subtle, differences between formulations when trying to optimize treatment for their individual patients. A reasonable starting position is to titrate to an adequate morning response, as for many patients this will be followed by good symptom control across the rest of the day. If not, further adjustments in dose, a change of MPH formulation or change to a different class of drug may be required. Such an approach is likely to lead to improved clinical care and should result in fewer patients requiring to be managed on multiple medications.

## Competing interests

A Gagliano received conference attendance support and speaker’s fees from Lilly, Novartis and Shire. She is or has been involved in clinical trials conducted by Lilly and Shire. The present work is unrelated to the above grants and relationships.

A Pelaz served in an advisory role for Lilly, and has received conference attendance support or received speaker’s fees from Bristol-Myers Squibb, Janssen, Juste, Lilly, Otsuka, Rubio and Shire. He has been involved in clinical trials conducted by Janssen and Rubio. The present work is unrelated to the above grants and relationships.

T Banaschewski served in an advisory or consultancy role for Bristol-Myers Squibb, Develco Pharma, Lilly, Medice, Novartis, Shire and Vifor Pharma. He received conference attendance support, conference support or received speaker’s fees from Lilly, Janssen McNeil, Medice, Novartis and Shire. He is or has been involved in clinical trials conducted by Lilly and Shire. The present work is unrelated to the above grants and relationships.

D Coghill served in an advisory or consultancy role for Flynn Pharma, Otsuka, Lilly, Janssen, Medice, Pfizer, Schering-Plough, Shire and Vifor. He received conference attendance support, conference support or received speaker’s fees from Flynn Pharma, Lilly, Janssen, Medice, Novartis and Shire. He is or has been involved in clinical trials conducted by Lilly and Shire and has received research funding from Lilly, Janssen, Shire and Vifor. The present work is unrelated to the above grants and relationships.

M Doepfner served in an advisory or consultancy role for Lilly, Medice, Novartis, Shire and Vifor Pharma. He received conference attendance support, conference support or received speaker’s fee from Lilly, Medice, Novartis and Shire. M Doepfner is or has been involved in clinical trials conducted by Lilly and Vifor. The present work is unrelated to the above grants and relationships.

A Zuddas served in an advisory or consultancy role for Astra-Zeneca, Bristol-Myers Sqibb/ Osuka, Lilly, Lundbeck, Shire and Vifor Pharma. He received conference attendance support and conference support or received speaker’s fees from Lilly and Shire. He is/has been involved in clinical trials conducted by Lilly, Shire and Vifor. The present work is unrelated to the above grants and relationships.

The systematic review was funded by Shire AG. Shire AG provided funding to Caudex Medical, Oxford, UK, for support with writing and editing this manuscript.

## Authors’ contributions

AG, TB, DC, MD and AZ made substantial contributions to the conception and design of the systematic review, and interpretation of data; AP made substantial contributions to interpretation of data. All authors have been involved in revising the manuscript critically for important intellectual content. All authors have read and approved the final manuscript.

## Authors’ information

Dr Antonella Gagliano, MD, PhD is Assistant Professor of Research in Child and Adolescent Psychiatry in the Department of Pediatric Science, University of Messina–Policlinico Universitario G. Martino Hospital, Italy.

Dr Antonio Pelaz, MD is a Child and Adolescent Psychiatrist at Hospital Clinico Universitario San Carlos, Madrid, Spain.

Professor Dr Tobias Banaschewski, MD, PhD is Medical Director at the Department of Child and Adolescent Psychiatry and Psychotherapy, Central Institute of Mental Health, University of Heidelberg, Mannheim, Germany.

Dr David Coghill, MB ChB, MD is a Reader in Child and Adolescent Psychiatry in the division of Neuroscience, Medical Research Institute, Ninewells Hospital, Dundee, UK.

Professor Dr Manfred Doepfner, PhD is Professor of Psychotherapy and Chief Psychologist in the Department of Child and Adolescent Psychiatry and Psychotherapy at the University of Cologne, Cologne, Germany.

Professor Alessandro Zuddas, MD is Associate Professor of Child and Adolescent Psychiatry at the Department of Biomedical Science, University of Cagliari, and Director of the Child and Adolescent Neuropsychiatry Unit at Cagliari University Hospital, Sardinia, Italy.

David Coghill and Tobias Banaschewski are joint first authors.

Antonella Gagliano and Manfred Doepfner are joint last authors.

## Pre-publication history

The pre-publication history for this paper can be accessed here:

http://www.biomedcentral.com/1471-244X/13/237/prepub
