# Erratum to: Long-acting methylphenidate formulations in the treatment of attention-deficit/hyperactivity disorder: a systematic review of head-to-head studies

**DOI:** 10.1186/s12888-015-0581-z

**Published:** 2015-08-25

**Authors:** David Coghill, Tobias Banaschewski, Alessandro Zuddas, Antonio Pelaz, Antonella Gagliano, Manfred Doepfner

**Affiliations:** Division of Neuroscience, Medical Research Institute, Ninewells Hospital and Medical School, Dundee, UK; Department of Child and Adolescent Psychiatry, Central Institute of Mental Health, Medical Faculty of Mannheim, University of Heidelberg, Mannheim, Germany; Department of Biomedical Sciences, University of Cagliari, Cagliari, Sardinia Italy; Department of Child and Adolescent Psychiatry, Hospital Clinico Universitario San Carlos, Madrid, Spain; Department of Pediatric Science, University of Messina, Policlinico Universitario G. Martino, Messina, Italy; Department of Child and Adolescent Psychiatry, University of Cologne, Cologne, Germany

## Correction

After publication of this work [1], we noted that a figure reproduced from Lopez et al. [2], was revised in an erratum.

In Figure three (Fig. 1 here), the uppermost solid line represents placebo and the lower solid line (at 0.5 h), represents Ritalin LA® 20 mg capsule. In addition, labelling of the x-axis should read “0.5, 1, 2, 3, 4, 6, 8” hours.

**Fig. 1:**
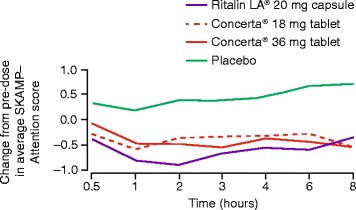
ᅟ
